# Assessing factors that influence perceived burnout in postdoctoral fellows and identifying recommendations to support their well-being

**DOI:** 10.1371/journal.pone.0344974

**Published:** 2026-03-17

**Authors:** Suzanne C. Harris, Emma Smits, Robert McGinity, Jacqueline M. Zeeman

**Affiliations:** 1 Division of Practice Advancement and Clinical Education, UNC Eshelman School of Pharmacy, University of North Carolina at Chapel Hill, Chapel Hill, North Carolina, United States of America; 2 UNC Eshelman School of Pharmacy, University of North Carolina at Chapel Hill, Chapel Hill, North Carolina, United States of America; 3 Division of Chemical Biology and Medicinal Chemistry, UNC Eshelman School of Pharmacy, University of North Carolina at Chapel Hill, Chapel Hill, North Carolina, United States of America; Johnson & Johnson MedTech, UNITED STATES OF AMERICA

## Abstract

**Background:**

While current evidence suggests rates of stress and burnout among healthcare professionals and graduate trainees are up to 50%, higher than the general population, there is a critical gap in the literature concerning factors which influence well-being among postdoctoral fellows in health sciences programs. This exploratory study aims to identify factors influencing well-being and burnout among these postdoctoral fellows and identify recommendations to improve their well-being.

**Methods:**

A two-stage sampling approach was used: (1) Purposive sample of postdoctoral fellows employed at a public university were recruited to participate in semi-structured focus groups to assess workplace factors which influence their perceived burnout and well-being and to solicit recommendations to improve well-being; (2) Stratified sampling was used to assign participants into focus groups by industry-sponsored or academic (non-industry-sponsored) positions to explore experiences that may be unique to these groups. Inductive coding and thematic analysis of Zoom transcripts were used.

**Results:**

Seven postdoctoral fellows participated in three focus group sessions, with two industry-sponsored fellows in one group, three academic fellows in one group, and two academic fellows in one group. Participants identified insufficient resources, difficult transition, and workload and program structure as factors contributing to their perceived burnout. Factors contributing to their well-being included reasonable supervisor expectations, personal and professional support, and resource support. Participant recommendations to improve well-being included institutional initiatives and resources, additional non-supervisor support, and workload strategies.

**Conclusions:**

This study expands upon the sparse literature on postdoctoral fellows by exploring factors that contribute to their perceived well-being and burnout, as well as provide suggestions to support their well-being. Findings contribute to the broader conversation of postdoctoral fellow well-being and burnout and informs the academy of focused strategies to improve their well-being and reduce burnout.

## Introduction

Burnout and stress among healthcare professionals is common, with up to 50% of clinicians and trainees reporting significant symptoms [1–6]. Among clinicians, burnout has been associated with worse patient outcomes, increased medical errors, and suicidal ideation [7–8]. In a recent consensus conference, leaders from several national pharmacy organizations recommended assessing factors which impact well-being [9]. Although well-being has no standard definition, scholars have highlighted the balance of positive and negative emotions, life satisfaction, and sense of autonomy as important components of well-being [10]. In contrast, burnout is defined as increased levels of exhaustion, demotivation, depersonalization, and a reduced sense of accomplishment [11]. People with lower well-being may be more susceptible to burnout and its personal and professional consequences [12].

Prior studies have explored factors influencing well-being in healthcare professionals, including physicians, pharmacists, residents, and students [3,13–16]. Specifically, studies focused on trainee populations identified relationships and social support as significant factors for positive well-being in both Doctor of Pharmacy (PharmD) and Doctor of Philosophy (PhD) students [14–17]. While studies with PharmD students identified personal traits as a contributing factor, studies with PhD students did not. Although these studies provide initial insights into trainee experiences, more information is needed to understand factors influencing well-being in trainee populations whose work primarily focuses on research, as these experiences may differ from those still engaged significantly in coursework and experiential learning within a degree program.

Available research conducted among those who work primarily in research, including PhD students, provide potentially unique factors affecting well-being, such as publication productivity, the role of the principal investigator (PI), curriculum and research stressors, work-life conflict, and financial burden [5,16]. Some research among doctoral students analyzed these factors according to specific subgroups. For example, Zhang and colleagues grouped PhD students by year in graduate school and found different factors significantly contributed to well-being between these subgroups [13]. Findings indicated advisor satisfaction was significantly associated with well-being only in 2nd year students, whereas sense of belonging was significantly associated with well-being only among 4th year students [13]. Another study grouped PhD students based on All-But-Dissertation (ABD) status (i.e., pre-ABD, ABD) [16]. While elements of curriculum and research stressors, lack of work-life balance, and financial burden were identified as burnout factors in both groups, unique aspects of these themes were highlighted between the groups. For example, Pre-ABD students emphasized expensive parking and stipend inequalities while ABD students emphasized uncertainties in program requirements and expectations around the timeline for program completion as specific stressors [16]. These results suggest that collecting and analyzing data by subgroups may be informative in identifying unique factors which impact well-being in postdoctoral fellows as well.

Although sparse, available literature among postdoctoral researchers has resulted in similar findings of reduced well-being as seen in clinicians [18]. Prior studies have also analyzed predetermined factors hypothesized to influence well-being in postdoctoral fellows, including relationship with their PI, publication productivity, and work-life integration [18,19]. Various quantitative methods have been used to measure well-being, including self-assessment of “life satisfaction,” Generalized Anxiety Disorder-7 (GAD-7), or the Maslach Burnout Inventory (MBI). While these studies assessed select factors hypothesized to impact well-being or burnout using quantitative measures, a gap exists exploring additional factors which may influence postdoctoral well-being, particularly for postdoctoral fellows in health professions programs.

While there are a growing number of studies in postdoctoral fellow well-being, the research exploring unique factors which influence well-being and burnout in postdoctoral trainees in health professions is primarily survey-based. Additional insight into factors influencing these domains would allow for optimized interventions to improve well-being. The objective of this qualitative study aimed to answer the following questions: (1) what factors positively affect postdoctoral fellow perceived well-being, (2) what factors negatively affect postdoctoral fellow well-being and/or cause perceived burnout, (3) what are recommended strategies to improve postdoctoral fellow well-being, and (4) what other thoughts or suggestions are important to this study? For the purposes of this study, burnout is defined as increased levels of exhaustion, demotivation, depersonalization, and a reduced sense of accomplishment [11]; well-being is characterized as the balance of positive and negative emotions, life satisfaction, and sense of autonomy [10]; and recommended strategies are ideas for action solicited from participants.

## Materials and methods

This study was submitted to the UNC Institutional Review Board and classified as exempt from review on July 2023 (IRB # 21–1629), with specification that no identifiable information (e.g., gender, age, race/ethnicity) be linked with participant responses. Participants provided electronic informed consent through QualtricsXM along with self-reported demographic data including gender, race/ethnicity, and advanced degrees held (PharmD, PhD, and/or master’s degrees). Participants consented to understanding that any data collected in focus groups transcripts would be deidentified to maintain confidentiality in any publications.

A two-stage sampling approach was used in this exploratory study: (1) purposive sampling of a public university in the USA (i.e., UNC Eshelman School of Pharmacy) that had previously conducted a quantitative well-being baseline assessment, and (2) stratified sampling into assigned focus groups. Results from the baseline assessment indicated that nearly half of the School community, including approximately 40% of postdoctoral fellows, were at risk for decreased well-being and burnout—findings similar to many other institutions and disciplines [20,21]. Postdoctoral fellows from this purposive sample, regardless of confirmed degree of burnout or well-being, were recruited via email in September 2023 to participate in a 60-minute focus groups conducted via Zoom in October 2023. Stratified sampling of postdoctoral fellow was used to assign participants into groups stratified by industry-sponsored postdoctoral fellows or academic postdoctoral fellows (non-industry-sponsored) to explore experiences that may be unique to these groups. Academic postdoctoral fellowships complete all or the majority of their research time in the academic environment. Industry-sponsored fellows complete one-to-two-year fellowships, typically with research time split between academic and industry environments, and begin fellowships together with their industry-sponsored fellow cohort. Participants self-identified their status (i.e., industry-sponsored or academic) during study recruitment. The initial focus group was led by a faculty research team member to train a doctoral student on focus group methodology, who then led the remaining two focus groups. The strategy was utilized to reduce potential relational bias of faculty or peer member presence and to promote a free exchange of ideas among participants about the postdoctoral experience. Notably, the faculty member leading the initial focus group did not have working relationships with any of the participants.

A semi-structured focus group script (S1 Appendix) was reviewed by faculty and trainees for refinement to ensure validity and reliability prior to implementation. The focus group script was used to guide the discussion and assessed three main areas: factors positively influencing perceived well-being, factors negatively influencing perceived burnout, and recommendations to improve well-being in postdoctoral fellowship programs. The semi-structured approach included probes to further clarify and explore factors influencing postdoctoral fellow well-being and burnout [5,13,18,19]. Participants were also provided a post-focus group optional single-item survey, at which they could anonymously provide additional thoughts not shared during the focus group discussion. Participants were provided a small monetary gift card in exchange for their time and participation. Focus groups were recorded and audio was automatically transcribed by Zoom (Version 5.3.11). Transcripts were reviewed for accuracy and de-identified prior to analysis. Transcripts and post-group survey responses were analyzed using inductive thematic coding. Initial coding and codebook generation was completed by one researcher. A second researcher independently audited each transcript using the developed codebook. Coded transcripts were merged and analyzed for agreement. All applied codes were reviewed for agreement by researchers, and any new codes or discrepancies were discussed until consensus was reached or data saturation was achieved. Inter-coder agreement was above the accepted 80% threshold for qualitative data [22]. This process ensured validity of code interpretation and richer data analysis. Microsoft Excel on Teams (Version 1.6.00.4464) was used for all thematic analysis. Per the study IRB protocol, no identifiable information (e.g., gender, age, race/ethnicity) be linked with participant responses. Thus, exploring demographic identifiers, such as gender and age, as factors affecting well-being or burnout was beyond the scope of this study.

## Results

Seven postdoctoral fellows participated in three sessions: two industry-sponsored fellows in one group, three academic fellows in one group, and two academic fellows in one group. The majority of the participants identified as women (86%) and held a PharmD (57%) and/or a PhD (57%) degree. Forty-three percent of participants identified as Black, and 21% each identifying as Asian or White. Additionally, one participant responded to the optional, anonymous single-item post-group survey, which aligned with recommendations for resources specific to postdoctoral fellows who are parents.

### Factors Influencing Postdoctoral Perceived Burnout

The most common factors contributing to burnout identified by both academic and industry postdoctoral fellows included insufficient resources, difficult transition, and workload and program structure (Table 1). Related to the theme of insufficient resources, one postdoctoral fellow stated, “Sometimes you’re trying to track down [training and orientation documents] and can’t figure out where to find it or who owns it…[That] carries stress over outside of work.” *(Industry participant I1.1)* Participants from both groups also commonly noted the challenges of transitioning into the role of postdoctoral fellow, with one postdoctoral fellow explaining, “a little bit of imposter syndrome comes in, especially for me, [be]cause I just transitioned from being a student…that transition was a little bit rough for me in the beginning.” *(Academic participant A1.1)* Participants also noted significant workload in their role as contributing to burnout: “You have to like run much faster as a postdoc than you were as a grad student…as soon as you start, you’ve got to be productive in showcasing.” *(Academic participant A2.3)*

**Table 1:** Burnout themes and sub-themes rank ordered by prevalence.

All Postdoctoral Fellow Participants (n = 7)	Academic (non-Industry) Fellows (n = 5)	Industry-Sponsored Fellows (n = 2)
**Insufficient Resources** ^**#**^		
• Resources not connected ^#^ • Resources unavailable^#^ • Insufficient parking or transportation^^^ • Lack of parental support resources^^^	• Resources not connected ^#^ • Resources unavailable^#^	• Resources not connected ^#^ • Resources unavailable^#^ • Insufficient parking or transportation^^^ • Lack of parental support resources^^^
**Lack of Belonging/Inclusion** ^**^**^		
• Lack of/insufficient DEI^^^ • Lost trust in institutional DEI^^^ • Inappropriately managed DEI situations^^^ • Misaligned DEI representation ^^^ • Lack of diverse community^^^ • Implicit stereotypes^^^ • Prejudice or discrimination^^^ • Lack of belonging^^^	• Lack of/insufficient DEI^^^ • Lost trust in institutional DEI^^^ • Inappropriately managed DEI situations^^^ • Misaligned DEI representation^^^ • Lack of diverse community^^^ • Implicit stereotypes^^^ • Prejudice or discrimination^^^ • Lack of belonging^^^	
**Lack of Relational Support** ^**#**^		
Lack of personal support^#^ • Lack of supervisor support^^^ • Lack of peer support^^^ • Lack of coworker support^#^ • Lack of faculty support^^^ Lack of professional support^^^ • Lack of supervisor career support^^^ • Lack of institutional career support^^^ • Lack of international postdoc support^^^	Lack of personal support^#^ • Lack of supervisor support^^^ • Lack of peer support^^^ • Lack of coworker support^#^ • Lack of faculty support^^^ Lack of professional support^^^ • Lack of supervisor career support^^^ • Lack of institutional career support^^^ • Lack of international postdoc support^^^	Lack of personal support^#^ • Lack of coworker support^#^
**Lack of Respect/Value by Others** ^**#**^		
• Supervisor-postdoc power imbalance^#^ • Lack of respect^^^ • Lack of autonomy in work/research^^^ ^•^ Time not respected^#^ ^•^ Not feeling heard^^^	• Supervisor-postdoc power imbalance^#^ • Lack of respect^^^ • Lack of autonomy in work/research^^^ • Not feeling heard^^^ • Time not respected^#^	• Supervisor-postdoc power imbalance^#^ • Time not respected^#^
**Difficult Transition** ^**#**^		
• Transition to position^#^ • Transition to new institution^#^	• Transition to position^#^ • Transition to new institution^#^	• Transition to new institution^#^ • Transition to position^#^
**Salary/Benefits** ^**^**^		
• Insufficient salary/benefits^**^**^	• Insufficient salary/benefits^**^**^	
**Workload and Program Structure** ^**#**^		
• Unreasonable workload^#^ • Unreasonable supervisor expectations^^^ • Unexpected factors^^^ **•** Lack of flexibility in position^^^	• Unreasonable supervisor expectations^^^ • Unreasonable workload^#^ • Unexpected factors^^^	• Unreasonable workload^#^ • Lack of flexibility in position^^^

^^^Theme or subtheme unique to one subgroup; did not appear in all subgroups.

#Theme or subtheme present across all subgroups. Only themes and subthemes that achieved data saturation within the subgroup are reported.

Among participants in the academic subgroup, one uniquely identified theme was a lack of belonging or inclusion. Feelings of not belonging were related to the lack of diversity, equity, and inclusion (DEI), with one postdoctoral fellow stating, “the lack of diversity for me is kind of draining” and “when I look at the University as a whole, I feel out of place.” *(Academic participant A2.1)* A common related subtheme was a lack of trust in the institution to address DEI issues: “Until there’s a systematic change and the people that exist within the School, all of the [DEI] work that they’re doing does not matter, especially when you have people who are able to walk around unchecked.” *(Academic participant A2.1)* Poor salary and/or benefits were also an emerging theme specific to academic postdoctoral fellows, and one fellow stated, “And we can’t even negotiate for salary in academia. That’s a main drawback, and I’m surprised that in many universities, the postdoc salary has been raised. In [the University], [they] haven’t increased [salaries] a bit, so I was bit surprised and kind of irritated.” *(Academic participant A2.2)*

Academic postdoctoral fellows also more commonly expressed feeling a lack of respect or value by others, as suggested by comments such as “postdocs are kind of at the bottom of the totem pole” *(Academic participant A1.1)* and “I feel I’m not always heard, and that because if it’s a postdoc asking, it’s not urgent.” *(Academic participant A1.2)* Related to this, supervisor-postdoc power imbalance emerged as a subtheme more prevalent among this group, as one explained, “If we have a disagreement that we can’t work around, then it becomes adversarial. Then I know my career is over.” *(Academic participant A2.3)* While all participants also expressed a lack of relational support, academic postdoctoral fellows highlighted a lack of professional support, especially from their supervisor, as a contributing factor. One participant explained the professional harms from a lack of supervisor career support, stating “If you have a [principle investigator] that isn’t thinking about your next steps, the postdoc just becomes a waiting period until you’re able to get out.” *(Academic participant A2.1)*

Additionally, while insufficient resources and workload and program structure were reported to contribute to feelings of burnout by both groups, industry-sponsored postdoctoral fellows more commonly highlighted insufficient parking or transportation and lack of parental support as inadequate resources contributing to burnout. One postdoctoral fellow who was also a parent explained, “another thing that I thought was challenging coming in was being a nursing mother and trying to find support and resources and needing to plan my schedule around pumping times and finding lactation rooms that were always full when I needed them.” *(Industry participant I1.1)* Reflecting on factors of workload and program structure that contribute to their burnout, industry participants discussed unreasonable workload as well as inflexibility inherent to the position. One participant explained the effect of burdensome workload, stating “whenever I can’t get all my workload done during work hours, that’s definitely impacts how satisfied I am and my well-being.” *(Industry participant I1.1)*

### Factors influencing postdoctoral perceived well-being

While aspects of relational support, resources, and workload were noted as factors influencing postdoctoral fellow burnout, similar elements across these themes were reported as factors positively influencing well-being among both academic and industry postdoctoral fellow groups. In relation to reasonable supervisor expectations for work, project workload and flexibility were emphasized by participants in both groups, with comments such as “we don’t have these strict deadlines that put us under a lot of pressure” and “negative results are results, but of course not publishable, but I’ve never had any pressure on that side, and I really enjoy that.” *(Academic participant A1.2)* (Table 2) Another fellow elaborated, “There is kind of flexibility that I do have in my postdoc, that I probably wouldn’t have anywhere else…that flexibility affords me more time to be at home, which is where I get my positive wellbeing.” *(Academic participant A2.1)* Having adequate support also positively influenced their well-being, and this was emphasized as both relational support and resource support. Participants commonly expressed presence of professional and personal support across supervisors, peers, and coworkers. One postdoctoral fellow summarized, “I’m lucky to have the team that I do because I feel very supported by my supervisor and everyone in the team.” *(Academic participant A1.1)* When referring to resource support, one participant stated that “[The program] do[es] a good job of telling you the different resources and telling you different grants you can apply for.” *(Academic participant A2.3)*

**Table 2:** Well-being themes and sub-themes rank ordered by prevalence.

All Postdoctoral Fellow Participants (n = 7)	Academic (non-Industry) Fellows (n = 5)	Industry-Sponsored Fellows (n = 2)
**Reasonable supervisor expectations** ^**#**^		
Flexibility^#^ • Flexible schedule^#^ • Flexible deadline ^#^ • Promoting days off^^^ Reasonable workload expectations^#^ • Publication/Research productivity^^^ • Total hours per week ^^^ **•** Project workload^^^	Flexibility^#^ • Flexible schedule^#^ • Flexible deadline ^#^ • Promoting days off^^^ Reasonable workload expectations^#^ • Publication/Research productivity^^^ • Total hours per week^^^	Flexibility^#^ • Flexible schedule^#^ • Flexible deadline ^#^ Reasonable workload expectations^#^ • Project workload^^^
**Relational support** ^**#**^		
Personal support^#^ • Coworker support^#^ • Supervisor mentoring^^^ • Peer support^#^ • Supervisory team mentoring^#^ • Sense of belonging with peers^#^ Professional support^^^ • Peer networking^^^ **•** Faculty support/networking^**^**^	Personal support^#^ • Supervisor mentoring^^^ • Coworker support^#^ • Peer support^#^ • Supervisory team mentoring^#^ • Sense of belonging with peers^#^ Professional support^^^ • Peer networking^^^ • Faculty support/networking^^^	Personal support^#^ • Coworker support^#^ • Peer support^#^ • Supervisory team mentoring^#^ • Sense of belonging with peers^#^
**Resource support** ^**#**^		
• Connection to resources^#^ **•** Support for postdoc transition^^^	• Support for postdoc transition^^^ • Connection to resources^#^	• Connection to resources^#^
**Respect/Value by Others** ^**^**^		
• Working autonomously^^^ **•** Feeling heard^^^	• Working autonomously^^^ • Feeling heard^^^	
**Value of the Position** ^**^**^		
• Fulfillment in work^^^ **•** Good salary and/or benefits^^^	• Fulfillment in work^^^ • Good salary and/or benefits^^^	

^^^Theme or subtheme unique to one subgroup; did not appear in all subgroups.

#Theme or subtheme present across all subgroups. Only themes and subthemes that achieved data saturation within the subgroup are reported.

While lack of respect or value by others was a common theme contributing to burnout among both groups, when speaking to factors influencing well-being, only academic postdoctoral participants reported feeling respected or valued by others as factors that contribute to their well-being. They highlighted feeling heard by others and a trust to work autonomously as beneficial. For instance, one participant described how postdoctoral fellows, as part of the broader School community, were invited to meet candidates for positions at the School. The participant stated, “[the School] invited students and postdocs to go sit at the seminar and actually have lunch with the candidate…they actually asked for feedback,” and “it was nice to see that you were included.” *(Academic participant A2.3)* Another participant explained how trust by others to work autonomously influenced well-being as, “our supervisors are very understanding of our independence and our ability to kind of get the work done.” *(Academic participant A1.1)* In addition to feeling valued by others, the value of their position was also a contributing well-being factor for academic postdoctoral fellows. This was supported by sentiments around having a reasonable salary or benefits and fulfillment from their work. Specific to benefits of the postdoctoral position, one academic postdoctoral fellow stated, “I think the health insurance plan is decent for postdocs. I’ve worked in [other settings] before, and this is probably one of the better plans I’ve had.” *(Academic participant A2.3)* One participant summarized their fulfillment in work as stating, “…research is not straightforward. I felt like in a wonderful environment to progress research and results” and “I think that’s what made me very happy to go to work every day.” *(Academic participant A1.2)* While relational support was a theme for both subgroups, only academic postdoctoral fellows spoke to the importance of professional support as part of their well-being, while personal support was emphasized more by the industry-sponsored fellows. Using networking events as an example of professional support, one academic postdoctoral fellow shared, “it creates conversation, and then you get to chat with people and maybe can also create work links, but also social links.” *(Academic participant A1.2)*

While many of the factors influencing well-being for industry-sponsored fellows were also common to academic fellows, a few related subthemes were more commonly expressed in the industry group. For instance, industry fellows emphasized the importance of being connected to resources as positively impacting their well-being. As one industry-sponsored fellow explained, “I feel the most supported in my well-being…when I feel like I have a project to do, and I’m connected to the people that I know can help me do it. I have all the resources I need, or I know where to look for them.” *(Industry participant I1.1)* Also, a theme within reasonable supervisor expectations unique to industry fellows was project workload, with one industry fellow commenting, “I do remember one of the last project[s] I started working on, the PI just took that project without any questioning, because I feel she understood that maybe that would be overwhelming for me.” *(Industry participant I1.2)* Additionally, while relational support was a theme common to both groups, industry fellows emphasized personal over professional support as most positively influencing their well-being. One participant from this group stated that relationships with collaborators on a more personal level “definitely helps you feel like you’re not coming up against challenges on your own and left out high and dry” and “You can tell that the faculty and staff really care about what you want to do with your future…always made me feel very supported.” *(Industry participant I1.1)*

### Recommended strategies to promote postdoctoral well-being

Top recommendations for strategies to improve well-being among both academic and industry postdoctoral fellows included four core themes: additional institutional initiatives and resources, non-supervisor support, supervisor support, and workload strategies (Table 3). Across both groups, participants identified a third-party mentor as a form of additional non-supervisor support that would be helpful, either in arbitrating conflicts or in providing peer support. One participant explained their recommendation “to improve well-being: a third party, like [a] mentor … and out of your field. It can be anyone from [the profession], but just [someone] assigned to a postdoc just to check [in]. I used to have that in in my grad school at [another University].” *(Academic participant A1.2)* Another participant elaborated on the importance of a third-party mentor other than the PI, explaining, “I wouldn’t have felt very comfortable if [advice] was coming from my PI, or the director of the program, or anyone who’s in a position of authority,” and “I wouldn’t feel that I could be as candid with them about my needs.” *(Industry participant I1.1)* In connection with insufficient resources identified as a factor for burnout, participants commonly recommended strategies for institutional initiatives or making resources more accessible. However, the specific institutional recommendations and resources (e.g., parking, events for well-being, parental support) varied by group. Recommended strategies to improve workload included opportunities to work from home and more personal time away from work. As it related to remote work, one participant stated, “if you’re not actually doing any lab-based work, [working remotely] seems like a very reasonable request to make, in my opinion, and it really promotes a much better work-life balance.” *(Industry participant I1.1)*

**Table 3:** Recommended strategies to support postdoctoral fellow well-being themes and sub-themes rank ordered by prevalence.

All Postdoctoral Fellow Participants (n = 7)	Academic (non-Industry) Fellows (n = 5)	Industry-Sponsored Fellows (n = 2)
**Institutional Initiatives and Resources** ^**#**^		
• Add resources for parents^^^ • Add wellness resources^^^ • Add parking/ transportation resources^^^ • Designate an onboarding/ resource point of contact^^^ • Adjust hiring contract and/or position expectations^^^	• Add wellness resources^^^ • Adjust hiring contract and/or position expectations^^^	• Add resources for parents^^^ • Add parking/ transportation resources^^^ • Designate an onboarding/ resource point of contact^^^
**Non-supervisor Support** ^**#**^		
• Build peer connections^^^ **•** Designate a third-party mentor^#^	• Build peer connections^^^ • Designate third-party mentor^#^	• Third-party mentor^#^
**Supervisor Support** ^**^**^		
• Encourage managerial training for supervisors^^^ • Supervisors support postdoc engagement/ networking^^^	• Encourage managerial training for supervisors^^^ • Supervisors support postdoc engagement/networking^^^	
**Workload strategies** ^**#**^		
• Improve workload strategies^#^	• Improve workload strategies^#^	• Improve workload strategies^#^

^^^Theme or subtheme unique to one subgroup; did not appear in all subgroups.

^#^Theme or subtheme prominent across all subgroups. Only themes and subthemes that achieved data saturation within the subgroup are reported.

In addition to the suggestions above, academic fellows also specifically recommended additional supervisor support. In connection with a lack of relational support contributing to burnout, participants recommended strategies to address these factors such as trainings that could help supervisors be more supportive. Examples of suggested trainings for supervisors included an “educational program regarding [international trainee legal requirements] before hiring any international student who has a visa” and programs “just to be an effective manager or leader.” *(Academic participant A2.1)* As part of this theme, participants recommended supervisor encouragement for postdoctoral fellows to participate in engagement or networking events as strategies to support their well-being, with one academic fellow stating, “maybe packing them in one email and...the PI can suggest, ‘Oh, that looks good. Or here are opportunities for you’…it shows that they approved you to go.” *(Academic participant A1.2)* Also, recommended strategies for non-supervisor support was highlighted by academic fellows, such as additional opportunities to build peer connections. One participant explained, “There was a lot of effort on the university-level for [events celebrating postdoctoral fellows]. I would have liked to see the same in the [School], so I could meet people working in the [School].” *(Academic participant A1.1)* Academic fellow participants also suggested adding wellness resources as part of institutional efforts, such as a “nice little flyer, ‘Hey, [are] you struggling? Oh, you want to talk to someone?’ Or [simply identifying] who you can go to.” *(Academic participant A1.2)*

Industry-sponsored postdoctoral fellows suggestions aligned with overall recommended strategies, though this group emphasized some unique institutional initiatives and resources to improve their well-being. Specific to institutional resources, one industry fellow, through the post-focus group survey, explained how postdoctoral fellows who are parents particularly benefit from peer mentors and suggested a strategy to accomplish this through “an informal mentorship/pairing system between postdocs who are parents.” Similarly, this sentiment also aligned with recommendations from industry fellows in the focus groups for more institutional initiatives and resources for parents, such as “a refrigerator that could have been used, or another lactation room” for nursing. *(Industry participant I1.1)* Parking was another specific recommended strategy for institutional or University support within this group, with one participant offering, “even like sharing [parking] space…if some faculty member can just be at School in the morning, a postdoc can use that space in the afternoon.” *(Industry participant I1.2)*

## Discussion

While published literature has begun exploring factors influencing well-being among trainees in healthcare and allied sciences, this study fills a literature gap as it is one of the first to explore factors that contribute to postdoctoral fellow perceived well-being and burnout, as well as solicit recommendations to support their well-being. This is an understudied population that has been mostly limited to quantitative survey data collection. Through the use of semi-structured focus groups in this study, participants were able to share their experiences, perspectives, and engage in collaborative discussion on factors influencing post-doctoral fellow burnout and well-being. Notably, this approach allows for participants to elaborate on responses and allows researchers to identify factors which may not have been previously identified. This discussion aims to synthesize the factors identified through thematic analysis to provide insight into factors of the post-doctoral fellow experience that influence their perceived well-being, burnout, and/or both, and thus ultimately inform strategies and recommendations to support their well-being. Findings illuminate actionable recommendations to support post-doctoral well-being and reduce workplace factors that influence their burnout.

In addition to academic postdoctoral fellows common to biomedical or health science-focused fellowships, this study included industry-sponsored postdoctoral fellows. Industry-sponsored fellowships support postdoctoral training through partnerships between academic institutions and industry-sponsor, with fellows often having mentors and research projects in both settings. Industry-sponsored postdoctoral fellowships are prominent in pharmacy training and include over 30 academic institutions and 80 industry partners [23]. Our study provides insights into shared and distinct burnout and well-being themes for academic and industry-sponsored postdoctoral fellows.

### Synthesis of well-being, burnout, and recommended strategy themes

This study identified several factors contributing to perceived burnout among postdoctoral fellows in health sciences programs. Additionally, themes that have previously been reported in healthcare professionals broadly such as high-stakes environment, inefficient work environment, burden of non-clinical duties, inadequate teaching time, and additional leadership roles, were not identified in this study [1,3,21], though a few factors overlapped with those reported in healthcare professionals or graduate students. One theme which emerged from our study was how insufficient resources contributed to burnout, which was common to both subgroups and included a variety of subthemes such as not being connected to resources and a lack of resources for parents. Lack of or unawareness of resources to achieve work goals have also been reported as stressors in healthcare professionals, though the lack of resources related to transportation or parental resources are not commonly cited in studies with healthcare professionals [3,21], and it is likely accessibility to these resources would vary widely among programs. Another unique theme was difficulty in transitioning either to a new institution or to the new role of postdoctoral fellow, which had not been previously reported in the literature. While other themes varied between subgroups, one common factor influencing postdoctoral fellow burnout was workload or program structure. This finding aligns with published literature among healthcare professionals, PhD students and postdoctoral fellows in science, technology, engineering, and math (STEM), which have associated work-life conflict with burnout [3,6,19,21]. Although specific to the academic fellow subgroup, a lack of belonging was a significant factor identified as contributing to burnout. This also aligns with published literature finding a lack of belonging was associated with poorer well-being among PhD students [13]. While there was some overlap with previous findings in graduate students and healthcare professionals, distinct themes within postdoctoral fellows also surfaced suggesting differences in workplace stressors for this group exists. Thus, future studies exploring how the emerging themes of insufficient resources for those with families or with transportation barriers, along with difficult transitions, may contribute to burnout are warranted.

Participants also identified a number of factors as contributing to their perceived well-being. While healthcare professionals reported involvement in patient care, peer and trainee education, and hobbies as factors that supported well-being [1], these themes were not reported by postdoctoral fellows. Common emerging themes included reasonable supervisor expectations, relational support, and resource support. Across these themes, the supervisor’s role in providing reasonable expectations and providing support professionally and personally aligns with similar findings in PhD students [13]. However, participants in this study uniquely highlighted the importance of personal and professional support from other community members, including their peers and coworkers. In connection with insufficient resources as a factor contributing to burnout, resource support – specifically being well-connected to resources – emerged as a factor contributing to well-being, which also aligns with findings in healthcare professionals [1]. Within the academic subgroup, participants spoke to the importance of support for postdoctoral transition for well-being, building upon their reflection that difficult transitions contributed to burnout. Additional research should further explore resources and support which may contribute to postdoctoral fellow well-being, especially during the transitionary period. There is limited overlap of well-being factors between postdoctoral fellows, graduate students, and healthcare professionals; thus, continuing to explore differences in factors contributing to well-being is warranted to better develop targeted solutions.

Several structural differences between academic and industry-sponsored fellowships may account for unique themes and subthemes in perceived burnout and well-being identified for the two groups. For example, unlike their academic counterparts, industry-sponsored fellows are required to complete coursework in additional to research, which may account for perceived decrease in flexibility. Industry sponsored fellows matriculate and graduate synchronously and participate in monthly forums to discuss emerging research topics and interact with industry leaders and alumni. In contrast, academic fellows onboard asynchronously and have fewer programmatic interactions, which may contribute to perceived lack of belonging. Moreover, industry-sponsored fellows typically perform research in both academic and industry settings and are supervised collaboratively by academic and industry mentors, likely enhancing both institution and supervisor career support. These insights may inform opportunities for enhanced strategies specific to the two groups.

During the same timeline as our study, the National Institutes of Health (NIH) Advisory Committee to the Director Working Group on Re-Envisioning NIH-Supported Postdoctoral Training synthesized feedback from a Request for Information (RFI) [24] and listening sessions [25] to assess major challenges faced by postdoctoral fellows and provide recommendations. Many themes identified in our study as major contributors to burnout, including insufficient salaries, inadequate DEI efforts, supervision-fellow power imbalance, and lack of personal and professional support, were also identified as major challenges by the NIH working group [25]. Close alignment of themes between the two studies emphasizes the interdependency of burnout and broad challenges faced by postdoctoral scholars. Our study also identified several unique burnout themes not addressed by the working group, including transportation and specific institutional resources for parents beyond parental leave policies.

This study also identified several recommendations made by postdoctoral fellows to improve well-being. Recommendations common to both subgroups included additional institutional initiatives and resources, non-supervisor support, and workload strategies. Although specific to one subgroup of fellows, additional supervisor support was another frequent recommendation. These recommendations mirror several factors identified in our study as contributing to burnout, such as a lack of relational support, insufficient resources, and workload or program structure. Additionally, some identified subthemes for recommendations are consistent with those previously outlined, including: providing appropriate staffing and resources for well-being, opportunities for community-building, recovery or wellness activities, and social support [26]. Also, the Advisory Committee to the Director Working Group on Re-Envisioning NIH-Supported Postdoctoral Training [27] and the National Postdoctoral Association (NPA) [28] have each issued recommendations for improving postdoctoral training. Similar to our study, both groups recommended clear definition of postdoctoral responsibilities, requirements for supervisor mentorship training, support for engaging in professional development, and non-supervisor support through peer connections and mentorship teams. In particular, expanding mentorship teams and strengthening cohort connections amongst academic fellows may address their sentiments to enhance well-being and limit burnout by reinforcing professional support and promoting a sense of belonging, respectively. While parental support was a complementary recommendation between our study and the NPA [28], the latter recommendations focused on extending family-friendly benefits such as Family Medical Leave, access to on-site childcare, and dependent coverage. Expanding on NPA’s recommendations, postdoctoral fellows in our study recommended a peer mentorship among parents who are fellows as well as ample parent-friendly resources such as refrigerators and lactations rooms for nursing parents. Our study identified an additional recommendation theme of workload strategies, beyond setting clear work expectations, healthy work environments, work-life balance workshops, or disconnecting after hours recommended by NPA [28]. Our study participants expanded on these recommendations to include flexible work models, such as ability work remotely on non-lab-based days to better support work-life balance.

While some similarities in recommendations exist with current guidance [26–28], prior research examining and evaluating the effectiveness of well-being interventions is mostly limited to behavior change or mindfulness courses for PhD students as helpful strategies to enhance well-being [29,30]. Recommendations from our study and other guidance documents extend insights beyond behavior or mindfulness courses, and programs may consider the implementation and measurement of additional interventions focused on providing resources, adjusting workload, documenting clear responsibilities, training supervisors as mentors, and increasing non-supervisor relational support to improve postdoctoral fellow well-being.

### Example strategies to support postdoctoral fellow well-being

Strategies have been initiated through our institution’s leadership or well-being action plans that align with broader calls to action in academic pharmacy or postdoctoral training [26–28] and address many of the participants’ recommendations to enhance well-being and to minimize burnout of postdoctoral fellows that other programs could adopt. For example, a new postdoctoral fellow orientation introduces school leadership, school well-being resources, including an embedded mental health counselor, and research and professional opportunities to support transition into a new position and new institution. The orientation also initiates community-building within the postdoctoral fellow cohort, which is maintained by an annual research retreat with an alumni panel, monthly social events for research trainees, and events during National Postdoc Appreciation Week. Professional support is strengthened by an Individual Development Plan and annual performance evaluation requirements that promote open communication and alignment of expectations and workload between postdoctoral fellows and their mentors. School postdoctoral development strategies are further supported by University initiatives led by the Office of Postdoctoral Affairs, which also align with contributing factors and recommendations from our study and recent postdoctoral training guidance [27,28] including: an online postdoctoral resource handbook, individual career counseling, postdoctoral fellow support groups that meet weekly to provide personal and professional support, postdoctoral writing groups, academic and industry-focused career development workshops, robust mentor training, and a regional postdoctoral research symposium.

Notably, post-doctoral salary and/or benefits was noted as a factor contributing to both burnout and well-being in academic post-doctoral fellows. Interestingly, at the time of the focus groups, the academic postdoctoral group had higher on average salaries compared to industry fellows at our institution; nonetheless, the finding at the time suggested that there was need for additional financial support. Notably, after the study focus groups were conducted, postdoctoral fellow salaries for both the academic and industry-sponsored groups have increased and become more comparable within the University as a result of guidance for salary determination based off the National Institute for Health National Research Service Award (NIH NRSA) [31] salary scale for doctoral trainees. While these are positive steps to support postdoctoral fellows, more research and exploration is needed to discover the continued impact of the stipend increase and other strategies on their well-being. These are a select example of strategies that other postdoctoral programs could consider to facilitate accessible resources, community-building, and professional support and financial support.

While this study addresses a critical literature gap, there are limitations to consider. First, this study intentionally leveraged qualitative methods using semi-structured focus groups to explore the experiences and perspectives of participants in relation to a spectrum of well-being and burnout. While postdoctoral fellows were identified as a group at risk for burnout, collectively, in a previous assessment, the data was not collected in way to link to participants’ qualitative responses to confirmed criteria for burnout; thus, strength of association between various factors and degree of well-being and burnout is beyond the scope of this study. Future studies could explore assessing burnout specifically in a confirmed burnout group, though because well-being and burnout exist on a continuum, these studies may introduce a biased sample when asked to address questions related to broader states of well-being. Due to the exploratory nature of this study, participants were from a convenience sample of volunteer postdoctoral fellows at a single institution; additional research that includes other trainees, fields of study, and/or institutions should be further explored to further enhance the generalizability of these findings. A notable strength of this exploratory study was intentionally focusing the sample size to allow for open dialogue and create a safe space for participants to express their thoughts. Future research should expand on this through studies of postdoctoral fellows across more institutions and in other health professional disciplines. Also, demographic data (e.g., gender, race/ethnicity) was not collected in way to link to participants’ qualitative responses. In an effort to avoid deidentifying data within a small sample, these associations were beyond the scope of this study. Also, parental status, age, length in program, or domestic or international status of participants was not collected as part of enrollment, and chronicity of factors expressed by participants towards either perceived burnout or well-being cannot be confirmed. As factors for stress persist, they could potentially lead to burnout; thus, future studies can expand on our work by assessing the chronicity of factors, and the other demographic variables, and their influence on well-being and burnout. Additionally, the timing of focus groups in Fall 2023 may not be representative of other times throughout the year. For instance, one study in graduate students found that indicators of well-being changed throughout the course of a semester [32]. Therefore, future studies may consider exploring factors at different times throughout the year to determine the consistency of these findings. Nonetheless, this study’s focus group approach provides initial insights into factors that influence burnout and well-being among postdoctoral fellows as well as recommendations to improve well-being, addressing a notable gap in existing literature.

## Conclusion

This study aids in filling a critical gap in the literature concerning factors influencing postdoctoral perceived well-being and burnout as well as provide recommendation for strategies to promote their well-being. Participants identified insufficient resources, difficult transition, and workload or program structure as factors contributing to burnout. Factors contributing to well-being included reasonable supervisor expectations, personal and professional support, and resource support. Participant recommendations to improve well-being included institutional initiatives and resources, non-supervisor support, and workload strategies. While some overlap of burnout and well-being factors exist in comparison to healthcare professionals and graduate students, unique themes emerged highlighting the different needs for postdoctoral fellows. This study contributes to the broader conversation of strategies to support postdoctoral fellows’ training experience and well-being across the academy and provide the groundwork for more research on this subject.

## Supporting information

S1 Appendix Focus group script. (DOCX)

S1 File Focus group transcript 1. (DOCX)

S2 File Focus group transcript 2. (DOCX)

S3 File Focus group transcript 3. (DOCX)

S4 File Codebook definition and Themes-subthemes. (XLSX)
