# Correction: Assessing factors that influence perceived burnout in postdoctoral fellows and identifying recommendations to support their well-being

**DOI:** 10.1371/journal.pone.0347250

**Published:** 2026-04-14

**Authors:** Suzanne C. Harris, Emma Smits, Robert McGinty, Jacqueline M. Zeeman

The third author’s name is spelled incorrectly. The correct name is: Robert McGinty. The correct citation is: Harris SC, Smits E, McGinty R, Zeeman JM (2026) Assessing factors that influence perceived burnout in postdoctoral fellows and identifying recommendations to support their well-being. PLoS One 21(3): e0344974. https://doi.org/10.1371/journal.pone.0344974.

Table 1 was uploaded incorrectly. Please see the correct Table 1 below.

**Table 1:** Burnout Themes and Sub-themes Rank Ordered by Prevalence.

All Postdoctoral Fellow Participants (n = 7)	Academic (non-Industry) Fellows (n = 5)	Industry-Sponsored Fellows (n = 2)
**Insufficient Resources** ^**#**^		
• Resources not connected ^#^ • Resources unavailable^#^ • Insufficient parking or transportation^^^ • Lack of parental support resources^^^	• Resources not connected ^#^ • Resources unavailable^#^	• Resources not connected ^#^ • Resources unavailable^#^ • Insufficient parking or transportation^^^ • Lack of parental support resources^^^
**Lack of Belonging/Inclusion** ^**^**^		
Lack of/insufficient DEI^^^ • Lost trust in institutional DEI^^^ • Inappropriately managed DEI situations^^^ • Misaligned DEI representation ^^^ • Lack of diverse community^^^ Implicit stereotypes^^^ Prejudice or discrimination^^^ Lack of belonging^^^	Lack of/insufficient DEI^^^ • Lost trust in institutional DEI^^^ • Inappropriately managed DEI situations^^^ • Misaligned DEI representation^^^ • Lack of diverse community^^^ Implicit stereotypes^^^ Prejudice or discrimination^^^ Lack of belonging^^^	
**Lack of Relational Support** ^**#**^		
Lack of personal support^#^ • Lack of supervisor support^^^ • Lack of peer support^^^ • Lack of coworker support^#^ • Lack of faculty support^^^ Lack of professional support^^^ • Lack of supervisor career support^^^ • Lack of institutional career support^^^ • Lack of international postdoc support^^^	Lack of personal support^#^ • Lack of supervisor support^^^ • Lack of peer support^^^ • Lack of coworker support^#^ • Lack of faculty support^^^ Lack of professional support^^^ • Lack of supervisor career support^^^ • Lack of institutional career support^^^ • Lack of international postdoc support^^^	Lack of personal support^#^ • Lack of coworker support^#^
**Lack of Respect/Value by Others** ^**#**^		
• Supervisor-postdoc power imbalance^#^ • Lack of respect^^^ • Lack of autonomy in work/research^^^ • Time not respected^#^ • Not feeling heard^^^	• Supervisor-postdoc power imbalance^#^ • Lack of respect^^^ • Lack of autonomy in work/research^^^ • Not feeling heard^^^ • Time not respected^#^	• Supervisor-postdoc power imbalance^#^ • Time not respected^#^
**Difficult Transition** ^**#**^		
• Transition to position^#^ • Transition to new institution^#^	• Transition to position^#^ • Transition to new institution^#^	• Transition to new institution^#^ • Transition to position^#^
**Salary/Benefits** ^**^**^		
• Insufficient salary/benefits^**^**^	• Insufficient salary/benefits^**^**^	
**Workload and Program Structure** ^**#**^		
• Unreasonable workload^#^ • Unreasonable supervisor expectations^^^ • Unexpected factors^^^ • Lack of flexibility in position^^^	• Unreasonable supervisor expectations^^^ • Unreasonable workload^#^ • Unexpected factors^^^	• Unreasonable workload^#^ • Lack of flexibility in position^^^

^ Theme or subtheme unique to one subgroup; did not appear in all subgroups.

# Theme or subtheme present across all subgroups. Only themes and subthemes that achieved data saturation within the subgroup are reported.

## References

[1] Harris SC, Smits E, McGinity R, Zeeman JM. Assessing factors that influence perceived burnout in postdoctoral fellows and identifying recommendations to support their well-being. PLoS One. 2026;21(3):e0344974. doi: 10.1371/journal.pone.0344974 41843608 PMC12994809

