# Supplementary material for: Assessing factors that influence perceived burnout in postdoctoral fellows and identifying recommendations to support their well-being
Source: PLoS One. 2026 Mar 17;21(3):e0344974. doi: 10.1371/journal.pone.0344974 (PMC12994809; doi:10.1371/journal.pone.0344974)
Supplement: S1 Appendix — (DOCX) [file pone.0344974.s001.docx]

| **S1 Appendix. Semi-Structured Focus Group Script** | |
| --- | --- |
| **Time** | **Prompt** |
| ~5 minutes | Hello, my name is (Moderator Name) and I’m the moderator for today’s focus group discussion. During today’s focus group, we will discuss factors which influence your well-being as a postdoctoral fellow at the School as well as recommendations to improve your well-being.  This research project is entitled “Identifying Factors that Influence Well-being.” You received a copy of the informed consent when signing up, thus we will only briefly recap it now. This focus group is being recorded so we can obtain accurate data about what was said. All data collected will be kept confidential. We ask that you keep this discussion confidential, as well, for the privacy of your peers. Your participation in this focus group is entirely voluntary, and you may stop participating at any time. You do not need to answer any questions you don’t wish to answer. As I ask questions, feel free to respond not only to my questions, but to others’ responses during the discussion.  Before we get started, I would like to clarify a few terms for the purpose of the discussion today. When I say “well-being,” I mean judging life positively and having an overall state of contentment. When I say burnout, I am referring to feeling mentally exhausted accompanied by negative emotions about yourself or others and decreased motivation and perceived performance.  The School has begun assessing well-being among its community members, including faculty, staff, students, and postdoctoral fellows. Findings from these assessments have helped inform strategies to improve well-being at the School. As an extension of these assessments, this focus group study aims to identify School-based factors influencing postdoctoral fellow well-being and burnout and identify recommendations to improve well-being. |
| ~15 minutes | What factors positively affect your well-being and bring you fulfillment? |
| ~15 minutes | What factors negatively contribute to your burnout? |
| ~15 minutes | What recommendations do you have for strategies to improve postdoctoral fellow well-being at the School? |
| ~5 minutes | What other thoughts or suggestions would you like to share that you think would be important for this study? |
| ~5 minutes | This concludes our discussion for today. If there is more you would like to share, please use the survey link for the post-focus group Qualtrics survey included in the recruitment email you received. There you can anonymously provide any additional feedback and/or perspective not shared during our discussion today. Thank you all very much for participating in this focus group, your input is very valuable in helping to inform and support future well-being efforts. |
|  | |
