# Supplementary material for: Assessing factors that influence perceived burnout in postdoctoral fellows and identifying recommendations to support their well-being
Source: PLoS One. 2026 Mar 17;21(3):e0344974. doi: 10.1371/journal.pone.0344974 (PMC12994809; doi:10.1371/journal.pone.0344974)
Supplement: S1 File — (DOCX) [file pone.0344974.s002.docx]

00:06:40.100 --> 00:06:41.750

Moderator (she/her): Good morning.

00:06:42.930 --> 00:06:44.470

A.1.1: Morning, Moderator.

00:06:44.720 --> 00:06:52.410

Moderator (she/her): Having a brief glitch with my video. My zoom, I suppose, is still waking up this morning. There we go

00:06:56.170 --> 00:06:56.990

A.1.1: Hold on.

00:06:58.250 --> 00:06:59.530

A.1.1: Okay.

00:06:59.670 --> 00:07:05.700

Moderator (she/her): good morning, A.1.1. Good morning, A.1.2. How are you?

00:07:07.100 --> 00:07:23.750

Moderator (she/her): Wonderful? Hey,there, [blind]! I think this is everyone we were expecting this morning, so we'll go ahead and get started for today. I know some of you on the call, but maybe not everyone. So a few quick introductions.

00:07:23.750 --> 00:07:47.010

Moderator (she/her): My name is Moderator, and I'll be moderating today's focus group discussion. Emma Smits is also here on the call. She's a member of our research team and she will be observing, moderating a focus group today. So, A.1.1 and A.1.2, you are our 2 participants, and Emma is here to learn more about how you can moderate a focus group.

00:07:47.280 --> 00:08:01.930

Moderator (she/her): So during today's focus group discussion, we'll be discussing various factors that you feel influence your wellbeing as a postdoctoral fellow here at the school, as well as any recommendations that you may have to improve your wellbeing.

00:08:01.930 --> 00:08:17.800

Moderator (she/her): So I have some templated language that I will read to be compliant with our IRB, and then perhaps we can do a few just quick introductions. So everyone knows who the other people are that are joining us today, and then we'll dive right on it. Does that sound Okay?

00:08:18.290 --> 00:08:31.500

A.1.1: Sounds good. I can start. I'm A.1.1. I work at the [blind] in the [blinded] Division in the [blinded] school.

00:08:31.670 --> 00:08:36.939

A.1.1: And yeah, I just recently graduated from [the University].

00:08:37.220 --> 00:08:41.259

Moderator (she/her): Great thanks, A.1.1. A.1.2, would you like to introduce yourself.

00:08:41.780 --> 00:08:55.479

A.1.2: Yes. Hi, I'm A.1.2. I am a postdoc in the [blind] Lab as part of the center for [blind] . So part of the [blind] Division of the School of [blind].

00:08:55.690 --> 00:08:59.929

Moderator (she/her): Excellent! Welcome, A.1.2. [blind], would you like to introduce yourself?

00:09:01.560 --> 00:09:09.879

Emma Smits: Sure, my name is [blind], and I'm currently a [blind] candidate at the [blind].

00:09:10.610 --> 00:09:36.519

Moderator (she/her): Wonderful. And then I'm Moderator. I'm on faculty at the school in our [blind] division, and then also service, the associate director and our office of [blind]. So in that role I help lead all of our assessment and valuation efforts at the school, and through that I'm partnering with [blind], who's our-- the school's director of wellbeing and the PI on this study, and partnering as well with [blind], who is our RASP student that's assisting with this particular study. So I'm gonna go ahead and dive into some of the IRB language, and then we'll open it up for our discussion around wellbeing today. So this project is entitled Identifying Factors that Influence Wellbeing. Each of you received a copy of the inform consent when you signed up and this will only briefly recap a few of the points here this morning. This focus group is being recorded. So we can obtain accurate data about what was said.

00:10:10.810 --> 00:10:28.260

Moderator (she/her): Be confident that all data collected will be kept confidential. So we do ask that you keep the discussion itself, also confidential To just respect the privacy of your peers that may be joining today. Your participation in this focus group is entirely voluntary.

00:10:28.260 --> 00:10:42.339

Moderator (she/her): and you may stop participating at any time. You do not have to answer any questions that you don't wish to answer, and as I ask questions, feel free to respond not only to my questions, but to what others have responded to dur-- throughout the session.

00:10:42.960 --> 00:10:59.020

Moderator (she/her): As we get started, I'll clarify a few terms that we'll use interchangeably throughout the day. When I say, wellbeing, I'm referencing those instances where you're judging life positively and having an overall state of contentment.

00:10:59.190 --> 00:11:10.639

Moderator (she/her): When I say burnout, I'm referring to feeling mentally exhausted, accompanied by negative emotions about yourself or others, and decreased motivation and perceived performance.

00:11:11.320 --> 00:11:40.220

Moderator (she/her): As you may recall or be aware, this will begin assessing. Well, being in community members, including faculty, staff students and postdoctoral fellows. Findings from these assessments have helped inform various strategies to inform school well being efforts. And as an extension of these assessments, this focus group aims to study and identify school based factors that influence postdoctoral fellow well-being and burnout, and identify recommendations that you and others at- others who are post docs at the school may have to improve Wellbeing.

00:11:44.540 --> 00:11:56.880

Moderator (she/her): so with that said, we're gonna open it up and hear from you, A.1.1 and A.1.2, about what factors you feel positively affect your wellbeing and bring you fulfillment.

00:11:59.790 --> 00:12:07.409

A.1.1: For me. It's a lot about support, I think. At the [blind] School of [blind]. You can tell that like the faculty and staff really care about what you wanna do with your future. And I think that's really important for post docs, especially because it's a—it’s a temporary position. And so, being willing to be open to just talking to them about what they like, and like being willing to be open to putting on projects and things like that has always made me feel very supported.

31

00:12:40.890 --> 00:12:42.679

Moderator (she/her): Great thanks for that A.1.1.

32

00:12:43.300 --> 00:12:46.260

A.1.2: Um, On my side, I say, the the mentoring team has been awesome in my experience, like my PI and all the I’m working on in that in that center, and all of them are very supportive, and also they don't pressure people into working to goals. They encourage hard work, of course, and thorough work. But there's no strict deadline that can put people under a ton of pressure. They really guide us when we have obstacles, and they fully understand that it might happen, and understanding that research is not straightforward. So I felt like in a wonderful environment to progress research and results.. being um.. happy about it. And I think that's what made me very happy to feel happy to go to work every day.

00:13:42.770 --> 00:13:45.849

Moderator (she/her): Okay, great. So I'm hearing 2 kind of tenets in your.. in both of your responses is element of support by supervisors or those in a supervisory type position. And then A.1.2 I heard you, raising also this element of flexibility, perhaps with work .

A.1.2: Yes, Very important. I think in I listen, my research, that we don't have like these strict deadlines like that put us under a lot of pressure. And I really enjoy to be in this event.

00:14:12.480 --> 00:14:39.270

A.1.2: It's not that I'm not working is that the research and going the way it wants, and so the results, like the positive results, might not be there yet, but negative results are results, but of course not publishable so, but that I’ve never had any pressure on that side, and I really enjoy that, and that motivates me even more to do a better job. To not have that pressure that I used to have in the past. Maybe.

00:14:39.330 🡪 00:14:41.959

Moderator (she/her): Ok, Ok, excellent! A.1.1, how about your experience?

00:14:42.280 --> 00:14:52.359

A.1.1: Yeah. And just to add on to that, I think flexibility… For sure I do definitely have deadlines and things, but they're very- They're.. you… They're easy to meet. Um And I think people are very understanding. If you are not able to meet them. I don't think I've ever come across someone saying, you should been able to meet that deadline.

00:15:09.860 --> 00:15:13.290

A.1.1: Um.. I also think that it's very like individualized, they really like cater The postdoc, position to what you like. um, and what you're interested in what you're skills can add to um the team.

00:15:28.960 🡪 00:15:30.130

Moderator: Okay, great

00:15:30.690 --> 00:15:58.410

Moderator: A.1.2, does that resonate with you? Or have you had a different experience?

A.1.2: Can I add a negative point? In my case, I'm very happy in the position and my supervisory team. But friends that are not as happy and I don't know if you can do anything, or if my counsel can be heard. But I found that as a postdoc you mainly only deal with your direct supervisor, and you don't really have a certain party involved in problem solving. You know, like [[blind]], my friend, was very unhappy, and it's hard, like, I guided them towards my PI, but it's completely like voluntary that we don't have someone assigned that can help solve conflicts or disagreements. So I just hope, raising that might help put a new system in place, maybe, because my friend got real unhappy from his position. And you

00:16:27.730 --> 00:16:39.350

A.1.2: only have your colleagues and your PI, I guess. And you know, it's you against your PI. And in these types of conflicts I find a third party might be helpful. I hope that input

00:16:39.820 --> 00:16:57.000

A.1.2: might be heard.

Moderator: Yeah, no, that- that’s great . I appreciate you raising that observation-

A.1.2: And the PhD students that I have, have like the whole, like committee to help them so if they, you know they have the chair of the committee that they can go to and, um, I feel that

00:16:57.260 --> 00:17:08.470

A.1.2: we don't have that, as a PI we only have- As a postdoc We mainly have our PI to go to, or we have to find ourselves ways to. Also, I'm sure the school of pharmacy is here. But we don't really- like the higher ups. I don't really know them. And he doesn't either.

Moderator: Okay, like higher ups within the school, or?

00:17:12.849 --> 00:17:25.779

A.1.2: Yeah, I know a lot of people like we can probably contact them that would hear us. But it's just not the same when they don't know their direct situation, I guess.

00:17:26.069 --> 00:17:27.339

Moderator (she/her): Right. Okay.

00:17:27.420 --> 00:17:28.739

Moderator (she/her): so it sounds like..

00:17:28.960 --> 00:17:47.360

A.1.2: In contrast to some other models, The the post Doc, is a direct report up to the PI, and there's really not much else in terms of like like extra support system..

A.1.2: Yeah, like in case the relationship with the PI is not ideal, I find.

00:17:47.430 --> 00:17:57.809

A.1.2: unless we know like, I'm sure if you look for help we can find it. But it's not like there, for, like my friend, for example, is international and struggling a bit more to reach out to people. So he needs to have a lot of- and trust.

00:18:03.970 --> 00:18:04.810

Moderator (she/her): Yeah.

00:18:05.430 --> 00:18:16.290

Moderator (she/her): let me probe on a a element of what you just shared, A.1.2, which was, you know, that your friend kind of confided in you some of this information, and and you're raising these points forward

00:18:16.840 --> 00:18:29.200

Moderator (she/her): for both you, A.1.2 and A.1.1. Can you speak a little bit about any relationships with peers, and how that influences your wellbeing at the school or your burnout at the school.

00:18:32.360 --> 00:18:39.160

A.1.2: Yeah, like, I'm pretty close with other like the postdocs in my research centre, we have this

00:18:39.190 --> 00:18:44.409

A.1.2: like Postdoc Group, where we meet monthly. And so we get

00:18:44.720 --> 00:18:46.849

A.1.2: to know each other and get

00:18:51.490 --> 00:19:01.780

A.1.2: each other’s inputs on our research because we present it like monthly. And we do like this little social like having a a Thanksgiving pot luck. And I think that's very important, because everyone feels that belonging, too. And it's just nice to go to work. And have people just asking, how was your weekend, or how was your day?

00:19:22.390 --> 00:19:26.270

Moderator (she/her): Great thanks for that, A.1.2. A.1.1, what about you?

00:19:26.640 --> 00:19:30.110

A.1.1: Yeah, I can definitely say the same about having other post docs to interact with. I really liked the postdoc awareness week. I think that was really cool to be able to meet other Phds and like, see what everyone's doing and being able to kind of just enjoy yourself. Bring family and.. those kind of events really like up the well, being a lot. and then I think it's different when you're in kind of like a unique position in your postdoc. I think Phds can kind of like mingle amongst themselves, and then, like, there's like division postdocs that kind of

00:20:24.040 --> 00:20:32.770

A.1.1: have, like their own events and things like that. So I think that might be a little bit on the burnout side where

00:20:32.860 --> 00:20:40.609

A.1.1: it's hard to reach out, to Post docs that you're not very. You're not interacting with on a daily basis, or don't have

in the same division as you.

00:20:42.920 --> 00:21:01.990

Moderator (she/her): okay, might be some kind of social pockets that are.. Infrastructure is influencing for like better phrase, okay, alright. Well, on that notion, let's transition to burnout. So for each of you, could you speak a bit to factors that negatively contribute towards your burnout that are affiliated with the school.

00:21:05.170 --> 00:21:08.539

A.1.1: I can go first. I think someone actually said this to me. So it, I think, like post-docs are kind of at the bottom of the totem pole. So when like you set up meetings with people, I think, like we're the first to go if something comes up. So there's like a lot of rescheduling. And when I was a student like, I didn't realize this but the students are like the most important people in the building. so I think that kind of contributes to my burnout when, like, I kind of like schedule everything around like meeting other people and- And then you have to kind of like switch around your schedule, which is not that bad. But I think that might be a contributing factor.

00:21:57.730 --> 00:22:04.139

A.1.2: I'd say, maybe is this in between positions between students

00:22:04.170 --> 00:22:32.860

A.1.2: and PI's, and sometime there's a lack of recognition, and that makes it a lot more complicated. Sometimes I have to bring my PI in a conversation or in a deadline, because if I'm on the only one interacting with the other people like, I feel I'm not always heard, and that you know, because it's a postdoc asking it’s not urgent, when, if I bring my PI in, then immediately I get my answers.

00:22:32.950 --> 00:22:47.049

A.1.2: Which I think is unique to the postdoc position. Because you are you in charge of your project. You have, like, you know, the Pi is more helping you and supporting your project. But at the same time, collaborators like

00:22:47.110 --> 00:22:49.360

A.1.2: they don't know your name, and

00:22:49.560 --> 00:23:01.760

A.1.2: they don't really like they- Everyone's busy, of course, but you become the least of the priorities. Maybe that's so maybe you relate, A.1.1, to things like that.

00:23:01.800 --> 00:23:10.339

A.1.1: I also think like a little bit of like imposter syndrome comes in, especially for me, cause I just transitioned from being a student

00:23:10.380 --> 00:23:20.780

A.1.1: to like working with my teachers. And so it's it's hard to just act like “oh, they're my coworkers,” and like finding that transition was a little bit rough for me in the beginning, but still feel very supported.

00:23:29.160 --> 00:23:29.950

Moderator (she/her): Okay.

00:23:33.970 --> 00:23:39.100

Moderator (she/her): A.1.2, you froze for a moment. So I just wanna check in to see if you're gonna add anything to A.1.1's comments.

00:23:41.930 --> 00:24:08.100

Moderator (she/her): Okay, okay, I just went check to the- at least on my end, The video froze, so I just wanted to make sure. So, A.1.1, you were kind of just talking about the acclamation piece, and how that transition into your postdoc At least in the very beginning. There were some elements that were contributing. Perhaps your burnout, could you? You mentioned imposter syndrome? But could you speak a little bit more to kind of that acclamation and transition period?

00:24:08.410 --> 00:24:14.310

A.1.1: Yeah, of course. I think one of the thingsthat impacted my burnout was

00:24:19.030 --> 00:24:39.150

A.1.1: when, like everyone's kind of in their flow. And you're like a new position you kind of come in in the middle of the year, or it's not very like, Hey, you're gonna start and we're gonna train you. And it's it's it's just like you start. And you start working and just kind of have to

00:24:39.850 --> 00:24:45.589

A.1.1: like, ask a lot of questions and just get used to what people are doing already.

00:24:45.790 --> 00:24:48.060

A.1.1: so maybe that maybe not a lot of

00:24:48.150 --> 00:24:57.359

A.1.1: training. I don't know how people could train me for this role. But I think there's no like transition period. You just gotta get in there.

00:24:57.830 --> 00:24:58.610

Moderator: Okay.

00:24:59.430 --> 00:25:03.870

Moderator (she/her): A.1.2, what was your experience Like as you transitioned into your postdoc role?

00:25:04.190 --> 00:25:13.190

A.1.2: So I have a bit of a unique experience. I arrive in March 2021, so that was still like heavily impacted by Covid

00:25:13.360 --> 00:25:23.050

A.1.2: which I think was not the worst time of Covid. So people started to interact. But it was a bit difficult to arrive in a new place. But I got- I got put in touch with someone from the lab was very helpful. And- but then, as you said in the lab, everyone has their own research ongoing, and it's a bit hard to find your spot at the beginning.

00:25:38.430 --> 00:25:47.959

A.1.2: When you have to learn every single thing, even the simplest ones. And people are just in their normal, like. like works, work habits. So they don't really have extra time to show you around or show you even the smallest details which can be a lot more complicated. I find also, of course, depends on the personality of the people like

00:26:08.550 --> 00:26:19.759

A.1.2: I needed someone to show me exactly how to do things, because that's how I work. I wanna do. I wanna completely adapt to the new environment and do what everyone does but

00:26:19.830 --> 00:26:31.729

A.1.2: Some people are just showing you maybe less into detail things that give you a big line of like. “Oh, do it like that, it’s fine. Oh, do it the way you want is fine.” But

00:26:31.830 --> 00:26:37.099

A.1.2: I think like people were always happy to answer questions, which helped a lot

00:26:37.240 --> 00:26:46.760

A.1.2: in that weird period, um, which was what matter the most to me that I felt immediately comfortable, asking questions around.

00:26:47.950 --> 00:26:48.830

Moderator (she/her): Okay, great.

00:26:51.050 --> 00:26:53.880

Moderator (she/her): Alright. So.

00:26:54.140 --> 00:26:59.800

Moderator (she/her): What other factors come to mind, as it relates to things that have been contributing towards your burnout.

00:27:15.810 --> 00:27:23.680

A.1.2: I'm not sure. I mean honestly, everyone’s been doing a great job.

00:27:23.890 --> 00:27:40.660

Moderator (she/her): So let's talk a little bit more about kind of your workload and/or workload expectations; that’s come through a little bit. Could you reflect a bit on how your workload, or those expectations influence either your wellbeing or your burnout in your current position.

00:27:42.120 🡪 00:27:51.220

A.1.1: So like Mel- A.1.2 mentioned. I think we have a very flexible schedule, and I think that has really helped me with my wellbeing just in terms of when I wanna go in when I wanna work from home. What times I wanna do that I think- our supervisors are very understanding of

00:28:06.570 --> 00:28:13.700

A.1.1: our independence and our ability to kind of get the work done on our own time. And I think.

00:28:14.360 --> 00:28:22.730

A.1.1: coming from Pharmacy School, where we kind of didn't have much of a grip on our own schedule, this is like, this is like the dream.

00:28:25.200 --> 00:28:31.199

Moderator (she/her): okay? So flexibility in in terms of your own schedule and hours. Alright.

00:28:32.110 --> 00:28:33.940

A.1.2: I think for me, like the workloads been. yeah, as A.1.1 said, like there, there's a trust that we can get things done. And I think it's actually working to, not push people to produce more and more. So we don't feel as stressed. But I believe my lab is highly productive in the environment. And

00:28:59.330 --> 00:29:07.490

A.1.2: everyone is more at peace like they- They fully understand that there's stuff that comes up in our private life that makes that they have like to show up later or leave a bit earlier, and they are totally fine with that. And everyone still gets the work done and is highly productive without having all these stress of I don't know. Just arriving on time every day, or scheduling like out of work meetings

00:29:30.490 --> 00:29:43.419

A.1.2: which are usually mainly only work hours. So that removes a lot of stress. Or if you know, something happens at home, you have to get a contractor like they are fine with you working from home, or

00:29:43.430 --> 00:29:45.760

A.1.2: and that's very, very pleasant for work and as I mentioned, to be understood when researchers doesn't go the way we want. It's also

00:29:54.840 --> 00:30:14.430

A.1.2: very important, which I think sometime like connecting, as always, the case, that people look for good results and exciting results. This is what everyone's look for, but unfortunately, this is part of the results that are not the ones we expect, and not really the ones that will give us the big story. So

00:30:14.560 --> 00:30:17.919

A.1.2: to be understood on that point feels very important for me.

00:30:18.710 --> 00:30:32.099

Moderator (she/her): Okay, great. And you both started to bring up this element of work-life, integration a bit. Within kind of workload expectations. So could you speak a little bit more about work, life integration within your current role.

00:30:34.820 --> 00:30:52.000

A.1.2: So I am fully lab. Oh, sorry. I am fully lab based. So I'm doing chemistry. So I can't do that at home and working from home when doing chemistry is not possible. We're very limited on what we can write; there is always like reports, and

00:30:52.050 --> 00:31:02.509

A.1.2: you know, papers and stuff you can do. But it's really limited. And it's not gonna last long. So I have to go to the lab. But I really like this work-life balance.

00:31:04.780 --> 00:31:18.750

A.1.2: As long as I am productive, I don't have to do crazy hours, and it's um, If I want to go in in the weekend or stay later. It's my decision. There's no pressure on that. And also

00:31:18.840 --> 00:31:29.069

A.1.2: I like, as I said, like I’m fully understood. If even if I worked hard, the results are not positive. We're not leading to a big story.

00:31:29.180 --> 00:31:31.929

A.1.2: And at the same time, if I have something coming up in my personal life. So it's really free to just warn my PI like emailing them and being like, oh, I'll be working from home for like this morning, or- or I won't be like working because I have something that came up. But that'll compensate, or or I think I'm fine on my work like right now, that's also fine.

00:31:55.160 --> 00:31:58.350

Moderator (she/her): Okay, Great, A.1.1, you're gonna jump in as well?

00:31:58.970 --> 00:32:08.740

A.1.1: Yeah, I think I was gonna say the same thing. I think in my role, personally like, I've never felt the pressure to kind of work beyond like business hours. And if I do, that's my choice, like, A.1.2 said, and, um, no one is kind of like shamed for not like answering in the middle of the night. And I think that's pretty unique to this role because there's a lot of pressure on the faculty side. There's a lot of pressure on the students’ side.

166

00:32:35.870 --> 00:32:38.780

A.1.2: Great. Yeah. My supervisor’s been supporting me like to take longer holidays and to take this some days off.

00:32:47.090 --> 00:33:04.889

A.1.2: So that's also great to be supported in that I'm I come from [blind]. So when I go back I have to renew my visa, and it's so when it's long, and it's never been a problem like recently I got stuck a bit longer because of visas, and I felt supported, which is awesome like it was not a bother, or

00:33:07.090 --> 00:33:19.419

A.1.2: when I got, say, I got Covid as everyone else and I felt like it was fine to stay home and rest, and I felt like really supported and not like pressured to be like, oh, you should get to work creating more data, which is really nice, too.

00:33:24.380 --> 00:33:34.719

Moderator (she/her): Okay, great. Well, each of you raised a number of different factors, some that contribute towards your wellbeing, some that contribute to your burnout, and those are really helpful. So thank you for that.

00:33:34.910 --> 00:33:48.400

Moderator (she/her): I'd like to transition to recommendations that you may have, whether that is things that the school should continue doing, or perhaps things that the school could do differently to help improve postdoc. Well, being

00:33:48.620 --> 00:33:52.120

Moderator (she/her): so, talk to us about some of the ideas that come to mind for each of you.

00:33:53.660 --> 00:34:23.110

A.1.2: as I mentioned, to improve a wellbeing is a third party, like mentor like, and out of your field. Maybe like it can be anyone from pharmacy, but just assigned to a postdoc just to check, like I used to have that in in my grad school in the [blind]. It was someone from the school, but not directly affiliated to my project, and the role of that person was just like, “How are you doing? Are you happy? Are you meeting like the goals you have for yourself? Are you being heard?” And I think that was so important.

00:34:23.170 --> 00:34:28.900

A.1.2: And I think that's something I would love to see implemented. For the postdocs.

00:34:29.460 --> 00:34:31.070

Moderator (she/her): Okay, great.

00:34:32.239 --> 00:34:42.049

A.1.1: I think like peer to peer either mentorship or just being able to have events or things where you could

00:34:42.199 --> 00:34:46.550

A.1.1: like interact with other post docs, I think- would be helpful like. I would have loved to have met A.1.2. Outside of this call. But it's great to like hear

00:34:54.840 --> 00:34:57.399

A.1.1: the PhD. Side of things as well.

00:34:57.480 --> 00:35:00.620

A.1.2: I think also, yeah, maybe

00:35:00.840 --> 00:35:20.679

A.1.2: having like more of this kind of social events like, there's been some with like free food. But people usually come and pick up and leave. So not really creating too much link in the end, and everyone's bit a shy. So you come with your peers to, and you don't really mingle but I think events like maybe conferences just for the postdocs like if everyone shows up, not everyone but who wants shows a presentation or a poster. It creates conversation, and then you get to chat with people and maybe can also create like work links, but also social links. I think.

00:35:38.290 --> 00:36:06.630

A.1.2: I think that's an idea I can share. And I would like to see implemented. Also, I think it's just good to know what other research is going in the whole department I'm not sure even in the different divisions I interact with. I'm not sure I know too well, if we have this conference. But I don't think the PACE postdocs are involved in the pharmacy stuff. So so I never interact with A.1.1.

00:36:06.660 --> 00:36:08.030

Moderator (she/her): Yeah, okay.

00:36:08.070 --> 00:36:21.060

A.1.2: So sounds like there's an opportunity for connection, both from a social perspective, but also, perhaps, research collaborations or just research, inquiry and better understanding.

00:36:21.460 --> 00:36:40.350

A.1.2 (she/her): I think what we have in common is a good conversation starter.

Moderator: Okay. And it sounds like there's some current effort around some social post-doc gathering like you kind of mentioned that sometimes there's food. But you kind of come and you go, or you come with your social circle. So could you speak a little bit more about

00:36:40.430 --> 00:36:46.370

Moderator (she/her): current things that are working well, and then modifications or recommendations you have to improve that

00:36:46.620 --> 00:36:47.610

Moderator (she/her): further.

00:36:48.630 --> 00:37:07.790

A.1.2: The free food is definitely working well, like at least the problem and everyone's going, but not many people are staying and mingling, as I said, like. that's like you come with your friends and you grab food, and then, you know work’s calling back, or you just eat it with your friends. And that's

00:37:08.180 --> 00:37:18.530

A.1.2: I think we need a little push, maybe to interact with all this. I know it's made to do so. But in practice it's not that straightforward.

00:37:18.560 --> 00:37:20.130

Moderator (she/her): Okay. Alright.

00:37:20.870 --> 00:37:24.920

A.1.1: yeah. I definitely think there- there was a lot of- um,

00:37:25.160 --> 00:37:50.600

A.1.1: effort on like the university level for Postdoc Awareness week. I would have liked to see the same in the [blind] school, so I could like kind of meet like people that are working in the [blind] school. When I went to like the postdoc awareness like social events and stuff. That was a lot of Phds from like all over the university. So it's it's hard to connect that way.

00:37:51.130 --> 00:37:54.419

Moderator (she/her): Okay? So mirroring perhaps some of the efforts that the university have but scaling it within the school. A bit. Is that right? Okay. Okay.

00:38:03.410 --> 00:38:22.170

Moderator (she/her): A.1.2, I wanna come back to one of the recommendations you've raised a couple of times, which is the opportunity to build out some type of mentoring or team based infrastructure beyond just your immediate PI, and you, you indicated that you had a similar type of experience with your training in the UK.

00:38:22.170 --> 00:38:42.599

Moderator (she/her): And so you specifically mentioned having, you know, at least one person that's not necessarily there to guide you on the research side, but just more the person side. So could you speak a little bit more in terms of you know. Do you feel like one person would be sufficient, or a team or peer mentoring kind of approach, or just unpack that a little bit what would be helpful for you?

00:38:43.320 --> 00:38:48.630

A.1.2: In my experience it was just one person, and I think there was like, if the person was

able to take actions which I think, higher up like PI’s, are able to. It was very helpful. I have just one personal example, like I was working on a confidential project.

00:39:01.560 --> 00:39:20.339

A.1.2: and as a PhD student I got a little worried that I wouldn’t be able to do posters and conferences and talk about my work, and I raised that up because I tried with my PI, but you know, he like always got like, “oh, you still have many more years” like, and then I raised it in that little meeting I had like yearly, and immediately the person raised it, and I got heard, and my PI fixed it, and I think it was just wonderful. Because sometimes, yeah, that being like one to one, it's a you know.

205

00:39:32.940 --> 00:39:41.409

A.1.2: you can't really change the balance. But having a third party involved might like make the PI realize also that it's a real concern, or it's important to deal with it. Or I have, like my friends, experience. He got really frustrated and- hard to find the right support. So he had to go through my own PI just- and he didn't want my PI to take actions, and It was really just to be heard and be guided from a different point of view. Like, “am I right to get upset, or is it the PI that is right?” And you know, in this one to one situation, the PI’s always win. Unfortunately, because we don't have much power. So I'd say one person that has ability to take actions, or that would be heard by your PI would be the best either from the school of Pharmacy or either from the PI teams. But I don't think peers would be very helpful, because I suppose, postdocs, unfortunately, we're not always heard.

00:40:43.010 --> 00:40:48.710

Moderator (she/her): Okay, that's helpful. A.1.1, what's coming up for you? Hearing A.1.2's reflection.

00:40:48.840 --> 00:40:58.909

A.1.1: Yeah, I was just thinking, like, I'm lucky to have the team that I do because I feel very supported by my supervisor and: everyone in the team. But I can't imagine like what it would be like if I didn't get along with my with my supervisor directly, because I- I only work with her or or I mean I do work with other people. But she is, I main contact. And

00:41:17.170 --> 00:41:21.270

A.1.1: yeah, I think like a tiered or like men- tiered like set up of mentorship might be useful for people that have trouble with just one person. And even if there's no conflict, I suggest also having someone checking and that I don't know.

00:41:37.310 --> 00:41:55.749

A.1.2: You're working normal hours, and you're taking care of your health. And you, I don't know this kind of basics. So just someone asking how you doing like, are you reaching your professional goals? And are you like happy in your position? Is there anything you have concerns about what's just great and like.

00:41:57.240 --> 00:42:09.079

Moderator (she/her): Okay, great A.1.1, you mentioned, your experience might be a little bit different. You mentioned this notion of a team. Could you. dive into that little bit more when you say, team. What does that look like for you?

00:42:09.600 --> 00:42:14.480

A.1.1: So I work in cipher. And so we have the director, this associate director and assistant director. So they make up the entire team. And I work on different things with different people within the team. And so I think that is a helpful way to like kind of have more people check in on you. I mean, I work, I mean, I have meetings with you a lot, Moderator, and those are always helpful to just like check in with someone about what you're doing and like, if they're interested in working with you. I've been able to have that like ability to set up like faculty meetings with people that are associated with our team and have that kind of way to like check in with more people.

00:43:06.530 --> 00:43:09.070

Moderator (she/her): Okay. Great. Alright.

00:43:09.890 --> 00:43:24.869

Moderator (she/her): well. Aside from kind of building out. A team based approach or just expanding the direct report infrastructure. What other recommendations do you have that the school could consider to help improve postdoctoral wellbeing

00:43:26.380 --> 00:43:31.839

A.1.2: maybe increase our salaries?

A.1.1: That would be great.

00:43:36.460 --> 00:43:45.500

A.1.2: yeah, I think these days it like everything is becoming more expensive. But the salaries- I know it's NIH based, but

00:43:46.040 --> 00:43:47.500

A.1.2: that'd be great.

00:43:47.570 --> 00:43:54.389

A.1.2: Even our benefits in our health insurance plans keeps like becoming worse and worse.

00:43:55.660 --> 00:44:05.250

Moderator: worse and worse, in terms of expense, or worse and worse, in terms of coverage? Could you unpack that saying?

A.1.2: Each time I check like they send us updates. And I don't know. There were a big discussion that there were too much cost. So they had to change the plan. And basically we got less coverage.

00:44:13.260 --> 00:44:15.690

Moderator (she/her): Okay. alright.

00:44:17.980 --> 00:44:23.539

A.1.2: but that's one of the good thing of postdocs is that we have the health insurance included.

00:44:24.030 --> 00:44:29.759

Moderator (she/her): Okay. so salary and benefits it sounds like are helpful, but perhaps could be improved.

(A.1.2 and A.1.1 nod).

00:44:30.220 --> 00:44:31.910

Moderator (she/her): Okay, alright.

00:44:34.060 --> 00:44:37.100

Moderator (she/her): What other recommendations come to mind for each of you?

00:44:42.170 --> 00:44:43.789

A.1.2: Definitely more- I'd like events that gather the postdocs because there's definitely a sense of belonging. When we meet with other postdocs.

00:44:53.530 --> 00:45:06.809

A.1.2: We're in the same boat, and it's it's just always good opportunities to be able to chat and share experiences and compare how everyone's doing. And I think it's very valuable. So I think the school of pharmacy has done an okay job at organizing things, but maybe more things, or or just organize slightly differently, so that maybe I know that it's more expensive. But lunches with time to sit down, I usually like bringing more mingling than just picking up a coffee or like that's my experience from the university-wide events. Um or the- As I said, the work related ones are also nice.

00:45:49.230 --> 00:45:58.870

A.1.2: I would add, for developing I like everything that's related to Yoga, my mindfulness, and there's been a couple of events. But- or the postdocs for the- with all this extra wellbeing event of the business of the well being fair, and I really appreciate all of these. I attend them. I think tomorrow I'm going to the meditation thing, I think, is cool of pharmacy, but it's in Marsico on the seventh floor. And all of that I really appreciate to so keep it working on these ones. But I know that’s not everyone like when I ask people to come with me to meditation? No, you really- not everyone enjoys it.

00:46:37.500 --> 00:46:44.790

A.1.2: but a variety of this kind of events that allows us to have a break in the day. I think I would enjoy them.

00:46:45.860 --> 00:46:48.180

Moderator (she/her): It sounds like you're supported by your supervisor in terms of engaging in those activities.

A.1.2: Yes, I am, yeah, As I said, we have flexibility, and as long as

there's some proof that I've been working hard, we could.

00:47:04.830 --> 00:47:07.409

A.1.1: Yeah, I think the encouragement sorry.

00:47:07.440 --> 00:47:15.160

A.1.1: Go ahead. The encouragement from our supervisors to just have a well balanced life, is it?

00:47:15.580 --> 00:47:19.169

A.1.1: It might just sound like a conversation, but it really does stick.

00:47:19.550 --> 00:47:20.400

Moderator (she/her): Okay.

00:47:21.830 --> 00:47:28.019

Moderator (she/her): Alright. Okay, this has been great. Kind of to the the social piece,

00:47:28.380 --> 00:47:35.110

Moderator (she/her): what I was hearing you raised A.1.2 and and A.1.1, you wanna check in to see if this resonates with you one. You're enjoying the different

00:47:35.750 --> 00:47:50.709

Moderator (she/her): activities in the variety of activities perhaps, that have been organized. There's ways that they could be improved so finding ways, for like of a better phrase, draw people in and have them stay, and perhaps an intentional effort to design it so that you could get to know those that are in the room rather than maybe those that you enter the room with. Only.

00:47:56.890 --> 00:48:02.699

Moderator (she/her): A.1.1, does that resonate with you as well, or anything that you would add or advice differently?

00:48:03.190 --> 00:48:07.920

A.1.1: I would agree with that. I think. That'd be very helpful.

00:48:08.010 --> 00:48:14.379

A.1.1: It would be cool to have, like the faculty and staff also like put in the effort to kind of get some of the postdocs as well. I think there is something in the [blind] Division like in the works, or I'm not sure about like a postdoc meet.

00:48:27.870 --> 00:48:35.509

Moderator (she/her): could you? That's that's helpful to me. Could you say a little bit more about the opportunity for faculty and staff to get to know the postdocs more?

00:48:35.930 --> 00:48:37.860

A.1.1: Yeah, I think.

00:48:38.130 --> 00:48:50.629

A.1.1: that really helps us kind of network a little bit better when there's like more social events rather than us like going to a faculty and having a meeting, and sometimes that kind of feels forced and like. We know how busy everyone is, and it's it's hard to like kind of for their time to just talk to you.

00:49:03.560 --> 00:49:04.380

Moderator (she/her): Okay. A.1.2, what are your thoughts and reactions to that?

00:49:08.690 --> 00:49:13.100

A.1.2: I think that's a very good suggestion. I don't think I really have. like postdoc-specific opportunities to meet with all the PI in the school of [blind], like, when people talk about PI’s like these rotation students, you don't know, like, I don’t even know who that is. When I- I know, like for my division we have for the pharmacy side. We have like this big retreat, but it includes everyone like the grad students that are maybe at the center of this type of events. And

00:49:37.110 --> 00:49:43.270

A.1.2: of course there's a part for the postdocs, but it's it's a massive event. So it's not really

00:49:43.810 --> 00:49:53.759

A.1.2: I don't know. I don't feel like I have a chance to talk to the. So the faculty and staff, like everyone, is also busy, like kind of judging, chatting with

00:49:54.120 --> 00:49:58.190

A.1.2: future collaborators and things like that.

00:49:58.660 --> 00:50:11.600

A.1.2: and I'd like to also add that on your comment that I think my PI, and I guess A.1.1 seems also to be the case. But are supporting us attending all these events. But

00:50:11.870 --> 00:50:21.039

A.1.2: I don't think it’s the case through all the school of pharmacy, and I think it'd be nice. I don't know if it's because they're not aware of it or not, but

00:50:21.310 --> 00:50:32.590

A.1.2: to have a reliable system of spreading the information. And I know a lot of my peers for every. I don't know what reason they don't receive the emails about all of these.

00:50:32.900 --> 00:50:46.729

A.1.2: So the mailing list, I see, is missing a lot of people and I'm not sure why, but just making sure, maybe everyone's included. Because if you don't hear about the events, there's no chance you will attend.

00:50:47.130 --> 00:50:51.530

A.1.2: and to have maybe the PI forwarding themselves.

00:50:51.930 --> 00:51:01.590

A.1.2: Some of the information would be also helpful, as it shows that they approved you to go. Not. Of course there's a lot of events and stuff. But

00:51:01.970 --> 00:51:15.689

A.1.2: I don't know. Maybe packing them in one email and be like the PI can suggest like, Oh, that looks good. Or here are opportunities for you, or just making sure everyone receive at least these emails.

00:51:15.840 --> 00:51:19.459

Moderator (she/her): Yeah, okay, great. That's a great suggestion, A.1.2.

Okay.🡪 alright.’We're starting to wrap up on our time. So as we conclude, what other thoughts or suggestions would you like to share that you feel would be important for this work.

00:51:34.120 --> 00:51:46.170

A.1.1: I think, like A.1.2 said. Like all the initiatives that wellness has been taking in- in the Pharmacy school has been like leaps

00:51:46.280 --> 00:52:03.439

A.1.1: with from like when I started. I think it did start like as I was starting. But it's been really helpful, and you can- You can see what is important to the Pharmacy school, because they're putting a lot of effort into the wellness initiatives.

00:52:04.380 --> 00:52:11.329

A.1.2: Add also sharing a bit more the resources for people struggling.

00:52:11.620 --> 00:52:25.029

A.1.2: I know I've attended recently, like the mental health first aid course. And they gave us, like all these resources in case, someone's really struggling with mental health. But I think also for lighter issues like to just know who to talk to or who to reach out for would be helpful. And I- I believe that could be a nice like email every now and then, or just also printing stuff like paper printing and putting them around the whole school of pharmacy like.

00:52:43.150 --> 00:52:51.570

A.1.2: And this nice little fly is like, Hey, you struggling like, Oh, you wanna talk to someone or just know who you can

00:52:51.630 --> 00:52:55.080

A.1.2: go to I think might be a good Idea too.

00:52:56.440 --> 00:52:57.580

A.1.2: Oh, thanks.

00:52:58.170 --> 00:52:59.020

Moderator (she/her): Okay. (she/her): So enhance the visibility and accessibility. Perhaps the resources. Okay.

A.1.2: yeah, just knowing where they are. And I think you know it it just if someone struggling, they know which flyer to go to which email to go to and

00:53:18.530 --> 00:53:23.580

A.1.2: or like, which person in the school of pharmacy will be the most-

00:53:23.760 --> 00:53:27.730

A.1.2: the best person to help with any issue.

00:53:28.110 --> 00:53:29.210

Moderator (she/her): Okay, great.

alright. Any other thoughts or suggestions you'd like to share.

00:53:38.820 --> 00:53:43.369

A.1.1: I do wanna say thank you for including us in thisA.1.1: whole initiative and this research project. And I hope it goes well.

00:53:48.370 --> 00:53:49.190

Moderator (she/her): yeah.

00:53:49.370 --> 00:54:10.560

Moderator (she/her): we appreciate your participation. You are a valued member of our community. And so we certainly wanted to create space for us to be able to hear directly from our post docs what their experiences like and what their recommendations are. So we appreciate. Both you, A.1.2 and A.1.1, joining us today and sharing your perspective and your experiences.

00:54:10.640 --> 00:54:35.049

Moderator (she/her): This concludes our discussion prompts, for today. you may have noticed, but we did include a qualtrics link in the actual meeting invite. And this is intended- If other ideas come to mind that you'd like to share that you weren't able to share today, or perhaps come to you after we conclude our discussion, and I'll I'll drop that link in the chat as well. Just for ease. The this

00:54:35.300 --> 00:54:58.180

Moderator (she/her): qualtrics survey is anonymous, so feel confident that any feedback you provide or your perspective would not be identifiable. But we'll be sure to include that in any of our analyses and thematic work that we do. So with that said, Thank you both very much for participating in this focus group. Your input is incredibly valuable and really helping to inform and support our future well-being efforts. So thank you both.

00:54:58.590 --> 00:55:17.390

A.1.2: Thank you very much. It's very good to feel heard.

Moderator: Great. We appreciate you. So I hope you both have a fantastic rest of your day. And feel free to use that Qualtrics link if any additional ideas come up. Okay, thank you very much.

00:55:17.420 --> 00:55:22.679

A.1.1: Nice to meet you. I'll see you around. Bye, Emma, thank you.

00:55:23.210 --> 00:55:24.270

A.1.2: Bye.

00:55:27.950 --> 00:55:28.660

Moderator (she/her): Okay.
