# Supplementary material for: Assessing factors that influence perceived burnout in postdoctoral fellows and identifying recommendations to support their well-being
Source: PLoS One. 2026 Mar 17;21(3):e0344974. doi: 10.1371/journal.pone.0344974 (PMC12994809; doi:10.1371/journal.pone.0344974)
Supplement: S2 File — (DOCX) [file pone.0344974.s003.docx]

00:06:35.710 --> 00:06:37.030

Moderator: Hi, I.1.1!

00:06:39.970 --> 00:06:41.700

I.1.1: Hi, Moderator! Can you hear me okay?

00:06:43.430 --> 00:06:50.349

Moderator: Yeah, I can hear you. Thank you for coming in. I think we're waiting on one other person, so I'll give it a couple more minutes.

00:06:50.480 --> 00:06:51.930

I.1.1: Okay, sounds good.

00:07:38.020 --> 00:07:39.339

Moderator: Hi, I.1.2.

00:07:51.030 --> 00:07:52.619

Moderator: can you hear me All right? Hi, Can you hear me Okay?

00:08:20.030 --> 00:08:25.850

I.1.2: (Background Noise of another voice) “You'd like to write this down” I.1.2 Speaking: Yes, I can hear you. Please give me just one moment.

00:08:25.900 --> 00:08:27.429

Moderator: Okay, sounds good.

00:08:35.309 --> 00:08:37.609

I.1.2: Yes. Hi, how are you, I.1.1?

00:08:39.289 --> 00:08:41.009

I.1.1: I'm good. How are you doing?

00:08:41.140 --> 00:08:42.570

I.1.2: I'm fine.

00:08:45.330 --> 00:08:49.810

I.1.2: How are you, Moderator?

Moderator: I’m good. How are you?

I.1.2: I'm fine.

00:08:51.190 --> 00:08:55.859

Moderator: Okay, so-

00:08:56.870 --> 00:08:58.519

I.1.2: So, I had, like, issues with my internet earlier. But everything is okay now.

00:09:06.420 --> 00:09:20.619

Moderator: alright. Well, I don't think we have anyone else joining us this evening, so I'll go ahead and get started. And I apologize if it looks like I'm looking at different screens. There's just some information that I'd like to give you before we get started.

00:09:21.040 --> 00:09:27.470

Moderator: So my name is Moderator, and I'll be the moderator for today's focus group discussion. So today, during today's focus group, we will discuss factors which influence your wellbeing as a postdoctoral fellow at the school, and any recommendations you might have to improve your wellbeing. So before I get into some of the more specific language, if you all would like to just introduce yourselves, describe your role and kind of the basic- what are- what you do in your role?

00:09:57.260 --> 00:10:06.520

I.1.1: Yeah, sure, I can go first. My name's I.1.1. I'm a [blind] year industry-sponsored fellow, and so I'm a [blind] fellow sponsored by the company [blind], and I'm in my second year. So I spent the first year at [blind] paired with [blind] as my mentor, doing research on his clinical studies, and then moved on at the end of the year to be with my industry sponsored company, and I've been with [blind]. The last couple of months doing- working with their clinical science team. Did I cover everything?

Moderator: Yeah, you're good. Thank you.

00:10:41.650 --> 00:10:42.470

I.1.1: Okay.

00:10:43.510 --> 00:10:53.629

I.1.2: Okay. I am I.1.2, I'm a [blind] year [BLIND] fellowship. So I do [blind] for research. So I spent a first year at [blind]. And I was working with Doctor [blind] and Doctor [blind]. and right now moved to a company still here in [blind] in pharmacy. I'm also working on picking up a new project right now. I'm working mostly on the [blind]. This is like, the analysis which is needed to be done before we start. Monday. Were you able to hear me?

00:11:27.950 --> 00:11:30.570

Moderator: yeah, yeah, okay, thank you.

00:11:30.830 --> 00:11:48.089

Moderator: thank you. so you should have received a copy of the informed consent when signing up. So this is just gonna be a brief recap. This focus group is being recorded so that we can obtain accurate data about what was said. All data will be kept confidential. We do ask that you keep this discussion confidential as well for the privacy of your peers. Your participation in this focus group is entirely voluntary, and you may stop participating at any time. You do not need to answer any questions that you don't want to answer. And as I ask questions, feel- feel free to respond not only to my questions, but to others’ responses during the discussion.

41

00:12:21.000 --> 00:12:36.940

Moderator: and before we get started I would like to clarify a few terms for the purposes of the discussion today. So, when I say “wellbeing” I mean generally judging my positively and having an overall state of contentment. When I say burnout, I am referring to feeling mentally exhausted, accompanied by negative emotions about yourself or others, and decreased motivation and perceived performance. So the School has been assessing wellbeing among its community members, including faculty, staff, students, and postdoctoral fellows. Findings from these assessments have helped inform strategies to improve well, being at the School.

45

00:13:06.230 --> 00:13:20.519

Moderator: as an extension of these assessments, this focus group aims to identify school-based factors influencing post doctoral fellow well being and burnout and identify recommendations to improve Wellbeing. so with all of that said, what factors do you feel positively impact your well-being and bring you fulfillment?

00:13:33.860 --> 00:13:48.499

I.1.2: I- I will go ahead and answer. So coming to [blind] the first factor that improved and kept me positive was the flexibility of the mentor I work with. So I was- the mentor working very flexible in terms of like when I have to submit, when I have to meet with them, If I'm available or not to meet with them. If I can record any meeting that way, or would you like reschedule? Or if I can just like, if I'm not feeling prepared to that meeting, I can just cancel those meetings. It was very flexible in terms of those meetings. but at the same time also for me. but at the same time also. They were very encouraging in terms of like meeting goals.

00:14:36.080 --> 00:14:40.729

I.1.2: and also in terms of like presenting the- I was, I was not prepared to present, like the whole project at one time, just one part of the project, or one thing that I have work on is sometimes to- to reach the end goal of whatever I have to do before. Like each of those meetings.

00:15:03.260 --> 00:15:18.449

I.1.1: Yeah, I think some. I have some similar ones, too. Flexibility is a big, big one from my mentor. That really helps with well, being just in a general sense, I would say, like it's helpful when, like my mentor asks about things that are going on in my life so that we can work around big events or things like that. I I.1.2 knows this, But I had a baby right before starting fellowship and had a a newborn, so that has its own challenges, and my mentor was always like very accommodating when necessary, and so like. I think that really helped my wellbeing, because we would have discussions about like, what are the expectations, and how can we make sure that I'm meeting them, even when I sometimes have outside factors that we need to work around.

00:15:58.770 --> 00:16:11.749

I.1.1: I think that was a big one, and some other factors with wellbeing, I think, for me, was the opportunity to work remotely a couple of days a week. I think that was- just that's huge. I mean, if I don't, if I'm just gonna be sitting in the office like joining virtual meetings or like working

on projects, and I may as well do that for my home office and save time with commuting, and I think that was a huge factor for me, for feeling a little bit more like satisfied with my work-life balance And I just thought of another one, too. Oh, I love sharing an office with Co-Fellows. That was like a really positive experience for my well-being. Like getting to make, uh connections with fellows, and like have a little bit of that social aspect, as well as part of them like postdoc experience.

00:16:51.100 --> 00:16:59.550

I.1.2: Yes, I agree. And I want to add to that that also working in a lab where you have. We have like in our lab with [blind] lab we have students and also we have, I don't know. Like a postdoc, like Postdoc student. he was very helpful because most of the time when you have like issue with maybe a project or something you can really like reach out to them, instead of like trying to reach out to the PI directly. And that was easy to do. And because we have everyone reach out to all those people. And even like, just move and talk with them in the same office. So yeah.

00:17:42.650 --> 00:17:46.539

I.1.1: yeah, that collaboration is like, really helpful.

79

00:17:47.010 --> 00:17:59.090

Moderator: So what I'm hearing is a lot of what promotes your wellbeing is kind of the relationships that you have both with your peers and your supervisor. Does that kind of sound like what I'm hearing.

00:17:59.100 --> 00:18:13.170

I.1.2: Yes, and how also organize those collaboration like this supervisor. Maybe at the beginning of a meeting we got you to somebody, and that's how you will break that relationship. And the next time you will just go to that person instead of coming back and ask the question to your supervisor.

00:18:21.670 --> 00:18:22.330

I.1.1: Hmm.

00:18:22.770 --> 00:18:25.540

I.1.1: yeah, just definitely helps you feel like you're not coming up against challenges on your own and like left out high and dry. I think that's an accurate assessment.

00:18:37.600 --> 00:18:38.740

Moderator: Wonderful.

00:18:38.780 --> 00:18:44.899

Moderator: And I did want to just probe on one specific factor and ask about it.

00:18:44.990 --> 00:18:52.939

Moderator: whether workload or workload expectations. Affect either your well-being or feelings of burnout.

00:18:56.080 --> 00:19:26.189

I.1.1: it can. It's certainly do- Do you wanna go first, I.1.2? Okay, it certainly can. I mean. there's been periods of time, and I think this is true in in any job, but especially in a postdoc position where, like, if you're coming up on deadlines or you know, due dates, or a big presentation, or something like your workload goes up and it certainly starts to influence your work-life, balance, and my well being as well, because mine, I'm

00:19:26.220 --> 00:19:41.840

I.1.1: much less satisfied when I feel like I'm having my family time taken away. and so for me, that's a big factor like whenever I can't get all my workload done during work hours, that's definitely impacts how satisfied I am and my wellbeing.

00:19:42.620 --> 00:19:45.699

I.1.1: But that's also like cyclical. It's not like that all the time.

00:19:49.620 --> 00:19:53.050

I.1.2: Yeah, what I want to say for my personal experience is that working as a [BLIND], or sometime I would prefer even to work in the afternoon.

00:20:02.640 --> 00:20:10.650

I.1.2: The the only reason is that maybe that is your preferred time for me to- to work. but at the same time I feel more productive at that time. so I'm not going to complain about like working after hours. Do Say I would complain about- Maybe like so if I had, like many projects at the same time. And then I have to like start to jungle and present those project also. Maybe at the same time, I would be like, I will feel overloaded. But that did not happen, and I do remember even one of the last project I started working on the PI just took that project without like any questioning, because I feel like she understood that maybe that would be like overwhelming for me. And yeah. so I just feel like, Okay, I can walk away from that.

00:21:07.060 --> 00:21:11.230

I.1.2: But I- I am happy that the number of projects I worked on was really like, it has a number. Yes.

00:21:19.710 --> 00:21:20.370

Hmm.

00:21:25.010 --> 00:21:37.340

Moderator: okay. Well, thank you for all of that feedback. We're gonna just transition a little bit more generally about what other factors you think might be contributing to feelings of burnout.

00:21:46.620 --> 00:21:55.479

I.1.1: This is kind of a silly one, but honestly, whenever I was feeling the most burnt out last year was whenever I hadn't figured out likethe schedule with like the bus and the commute, and like there's it’s so like demoralizing to finish a long day of work, and then sit and wait for the bus to the park and ride, and like it doesn't show up when it's supposed to, and then just sitting in traffic.

00:22:13.810 --> 00:22:22.809

I.1.1: And like, I know, that's not something anyone can control. But you know, finishing your day and then having it take an hour plus to get home is just so frustrating.

00:22:25.670 --> 00:22:35.589

I.1.1: And I eventually figured it out like figured out a schedule that worked a little better for for me, based on factors, but I felt as the most burnt out whenever that was happening, for sure.

00:22:36.670 --> 00:22:45.380

I.1.2: Oh, yes, I.1.1, thank you for bringing that up. That was also one of the thing that I really think very, very stressful about that. Like to not be able to park like in a reasonable, reasonable area close to the school.

00:22:55.990 --> 00:22:57.220

I.1.1: Yeah.

00:22:57.540 🡪 00:23:01.950

I.1.2: because I feel like, first, I waste a lot of time just like to go from my house to the parking lot and then pick a- get a bus, and sometime I will wait for the bus for like 1 hour. and then get into the bus, get to work, and then the time to go back home. Wait another 1 h to get in on the bus.

121

00:23:18.030 --> 00:23:22.420

I.1.2: so that was very frustrating, and I don't know if something can be- could be changed due to this, to do- For the- the amount of space we have like at [blind], that might not change, but maybe, like, in terms of like buses or even like sharing space. like maybe I don’t know, if like some faculty member can just be at school in the morning a postdoc can use that space in the afternoon, stuff like that. I don't know. But it was very like stressful for me.

126

00:23:55.170 --> 00:24:19.450

I.1.1: Yeah, yeah, me, too. And especially once I was able to like, when I was training, I was having to come on site every single day for a long time, but by the time I was fully trained I only had to come on campus like maybe 3 days a week, and I could work from home, the other 2, and that like was an immediate shift and well being, and I just felt like so much more satisfied once I wasn't feeling like I was wasting 15 plus hours a week just with commuting.

00:24:23.380 --> 00:24:27.810

I.1.1: But I think some other ones I kind of liked when we would have, like our fellowship events. And I know it's a little different than like the academic postdocs’ positions. But when we have, like our weekly or not weekly, our like monthly fellows forums, or we have, like social events that are put on by the program that let us get to know our co-fellows and our directors and mentors and stuff. I felt like that was a nice way to feel more connected to the program. And like.

00:24:57.690 --> 00:25:05.930

I.1.1: if you're talking about well-being like I feel more satisfied and happier when I feel connected to the people that are involved in like the program around me. So that was something. I whenever, whenever it worked out and I was able to actually attend just them.

00:25:12.160 --> 00:25:36.659

Moderator: I do wanna pull out one piece of what you talked about earlier and kind of some of the challenges that you had in training, and maybe your transition to the role that you’re in would either of you say that there was any part- other part of your transition into your current role, or your training that might have affected wellbeing or burnout?

00:25:39.050 --> 00:25:47.049

I.1.1: at like, whenever we transitioned into the program, or once we transitioned like, into the second year away from being at [blind]?

00:25:47.730 --> 00:25:48.670

Moderator: Either.

00:25:48.930 --> 00:25:49.720

I.1.1: okay. You got anything I.1.2?

00:25:53.960 --> 00:26:05.529

I.1.2: yeah, for the transition into the program. It was at the beginning. I was a little bit lost in terms of like: really the commitment. but after that I was fine. But 1 point where I felt also lost is when we were transitioning from like was the first (*cannot make out audio)*. And at that point we- I realized that we have, like maybe 4 to 5 classes to take.

00:26:30.810 --> 00:26:35.220

I.1.2: And most of those classes were not really like in person classes. They were classes that were given last year, and 🡪they were given the previous year, and last year those classes were supposed to not happen. So we just have to like, listen to recording and try to teach ourselves. So I feel also overwhelmed to the point where I even did not listen to all those.

00:27:01.700 --> 00:27:07.929

I.1.2: I prefer to go in person on all the time and for me was like, Okay, if those classes are only offer once every like 2 years, I'm going to wait in my second year. I'm going to listen to those classes because I don't know. I- I have like a- a break down at that moment where I feel like, okay, I have to read all this. the the faculty members were available. But you have to maybe like, make an appointment for them to go and see them, and also be prepared with your question. And it was a little bit for me.

00:27:43.220 --> 00:27:50.849

I.1.2: I have a break down at that point. Yeah. And I feel very discouraged to go into those classes to- to read on my own. It doesn't mean that I would have not done that. If it was more structure, if it was like, maybe like a normal online class where we have like weekly assignments, weekly, readings, but it was like. Everything was just dumb to us. We have to. Yeah, everything was dumb to us at once, and that was that was it.

00:28:13.600 --> 00:28:17.210

I.1.2: And just because those class were not offered last year.

00:28:21.710 --> 00:28:37.199

I.1.1: I didn’t take as many classes as you did, since you're doing all the [BLIND] ones. But, there were a couple of challenges I had during training that made the transition a little hard for me. I'd say, like one of the big ones wasI didn't have a lot of overlap with previous, like members of the lab that I was kind of joining.

00:28:49.460 --> 00:29:01.450

I.1.1: And so there's a lot a lot of like training documents and and things that outline the information I need to know. But there wasn't a lot of like face to face time with people that really knew what was going on, and I don't actually think that there's much we could have done about that, because there was supposed to be. And we had some people like unexpectedly leave the lab.

00:29:15.130 --> 00:29:29.130

I.1.1: so I- I don't think it was anyone's fault, but it does make it challenging whenever it feels like- Sometimes you're trying to track down information and can't figure out where to find it or who owns it, or who knows what and that kind of like carries stress over outside of work. Cause I feel like I'm having to spend all my time reading up on training documents and things like that.

00:29:34.120 --> 00:29:44.170

I.1.1: But another thing that I thought was challenging coming in was being like a nursing mother and trying to find support and like resources at the school and needing to, you know, plan my schedule around like pumping times and finding lactation rooms that were always full when I needed them. And there are a lot of good resources available. But I- I had a hard time finding them sometimes and integrating it into my schedule, and I- I know that, like some of the people I ended up talking to were very supportive. But when you start in a place that's brand new, you don't know who you can talk to, and who you can trust, and who's gonna like view you differently if you need to be looking for accommodations for that kind of thing. So that was a source of stress on my well-being when I started out for sure.

00:30:34.340 --> 00:30:35.929

Moderator: Yeah, for sure.

00:30:40.760 --> 00:30:49.829

Moderator: Are there any other factors or things that you'd like to mention for either of you related to things or factors that impact burnout?

00:30:54.030 --> 00:31:02.779

I.1.1: in a positive way? Or should we talk like some of the negatives as well, or will we get into that more?

Moderator: Things that might be causing burnout.

00:31:05.520 --> 00:31:09.039

I.1.1: I guess one thing I haven't mentioned is I don't know like what the word is to put to it, but when, when when you get things sprung on you and you can't plan for them. They’re like, “Oh, you have this meeting. You have to go to today,” or like, “Oh, you have this big event that no one told you about that you have to now rearrange your whole schedule for to make happen.”

00:31:31.930 --> 00:31:34.959

I.1.1: that happens sometimes, and that is like contributes to burnout for me cause. You know I- I plan my- my day, my week, my month in advance. I think most people do, and so, whenever you now have to rearrange everything and like take away from personal time. I don't. I keep saying that, but that definitely contributes to burnout for me, and I get very annoyed.

00:32:01.370 --> 00:32:09.730

I.1.2: Yes. One other thing I want to add is I. This is this, the- the computer system that we have to use- when we- When we want to use the phone in order to go to meetings, to conferences. and for me it was so stressful to just like use the system because the system, Even after I have used that system for so many times, even as of today. it's still not clear what to do, how to do it. And what are the main information that you need to enter into the system.

191

00:32:40.530 --> 00:32:51.080

I.1.2: like? I do remember last year during the fall, there was one system. During the spring, It was different. How you have to enter your items! It was so different for what I did in the fall. and when I came this year I hope it's not going to be different.

193

00:32:55.490 --> 00:33:02.159

I.1.2: And also and also like sometime we like, for instance, for the hotel and the- the taxi and everything, we need to sometimes pre-pay pay using our own cards. And sometimes those hotels can go up to like a thousand dollars for like just like 4, 5 days. And and this can be very like difficult, for, like a student who has not planned financially to make that and be able to to- to get the money back in like- mine was at least 2 months after. Because

200

00:33:39.160 --> 00:34:04.450

I.1.2: the first thing, yeah, not because, I know. I know that if I entered the information right away, I would get the money one month after. But mine was always two months after, because the items that we needed the way it was entered. It was always like, very stressful to me, to the point where sometimes we just forget about it and say, Okay, I forget about it. And also sometime I will not have like cooperation with the the other company, because I do remember one time I have like, a rental car and those people like gave me like everything was in computer, and the system was in the compueter. And they told me that, okay, because of the system.

202

00:34:18.830 --> 00:34:35.180

I.1.2: I will not be charged certain amount of money, and then I was charged that on my card, and only at the last minute, maybe 2 months after. I got a response that okay, that money can be reimbursed, because anyway, I used that For the conference. And, I mean, it was just like. I mean, the computer systems still need for me to be like revised, because all the time I have to go and do something with that system, I have like. have a lot of stress.

208

00:34:55.900 --> 00:35:00.490

I.1.2: Yeah. And it can take me like this in order (gestures) to figure out one thing.

00:35:01.740 --> 00:35:03.929

I.1.1: Yeah, I had the same experience that when I was trying to find out. Hmm! Going to a conference. I think I wasted at least 2 or 3 entire workdays just trying to figure out the system so I could get reimbursed for my conference, and I'm like I have so much better work I could be doing. This is not contributing well to my productivity right now. But

00:35:25.420 --> 00:35:28.319

I.1.1: actually that kind of raises a point I was going to say about something I noticed over the past year for me, which is I came from a much smaller university for pharmacy school and for undergraduate- my undergraduate degree as well, and I find it like a sort I don't know. I just find myself getting burnt out trying to figure things out at such a big university like

00:35:52.060 --> 00:36:15.750

I.1.1: it's always been my experience that I can just walk into someone's office and ask them for help. Whereas at [blind], like there's a separate department for every single thing you might possibly need. And you I don't know where they are. I don't know where the buildings are. Nobody wants you to just show up at their office. They want you to go through the the portals to submit requests for every single thing that you could need, and everything about it feels so impersonal, and it kind of makes me just like I don't know. It just feels really stressful trying to figure out anything I need to do, because I don't know who to go to or who can help me? And ultimately, like, once you do find people, they tend to be very helpful. But-

00:36:37.070 --> 00:36:43.209

I.1.1: it's just yeah much, much bigger than I'm used to, and it sometimes kinda makes you feel a little bit lost in it all.

00:36:44.550 --> 00:36:50.950

Moderator: So what I'm hearing is sometimes it's hard to get connected with the right resources when you need certain support.

00:36:51.090 --> 00:36:53.180

I.1.1: Yeah, that's a good way of putting it.

00:36:57.350 --> 00:37:03.020

Moderator: I.1.2, Have you had any similar experiences or different ones related to that.

00:37:04.050 --> 00:37:10.830

I.1.2: I don't have the same experience with like the IT department, not for other departments. And- but I think that's finally it works. Because I, at 1 point, like I.1.1, as mentioned, I just decided to always walk to them.

00:37:24.150 --> 00:37:29.520

I.1.2: because, like most of the time, even when you submit, I submitted my request using the portal, and wait like many days, for my request to be answered. and then, I remember, then I will talk also with, One person, so I also have to see, [blind], and we also talk with a navigator which was very, very helpful.

00:37:41.560 --> 00:37:52.860

I.1.2: just so I walk.

00:37:53.000 --> 00:38:15.759

I.1.2: they, most of the time I find somebody. Yes, for sure. When you find somebody you are going to be helped right away. It's just like, if you don't find somebody like I have my phone. Finally, I get the number on my phone and say, “Okay, are you there today?” “Oh, no, I'm not there today. I'm working from home like, can you submit everything online?”

00:38:16.500 --> 00:38:31.839

I.1.2: “Okay, come tomorrow. Have a good day.” And this one, you you find somebody. Yeah, the person is going to help you right there. But the thing is that the process of finding that is that the process of getting to see somebody, the process of like

00:38:32.260 --> 00:38:38.340

I.1.2: So meeting, and then you don't get any response on time. I mean, I don't know what is on time. But sometime they say you have like 72 hours. Sometime, man, we go over the time that was mentioned. I don't remember even once I didn't submit a request. Somebody submitted on my behalf, and on my behalf I didn't have to to wait for like a week.

235

00:38:58.790 --> 00:39:01.810

I.1.2: I mean stuff like that will be very stressful mostly when you have like those software you want to- you want them to run on your computer. And then. you have to wait. You just have to. You don't have anything else to do just to wait. Like.

238

00:39:17.500 --> 00:39:18.570

I.1.1: yeah. yeah, definitely, I think that's a big, like big source of like. Oh, my gosh, I my words are escaping me, but I think I feel the most like supported in my wellbeing, feels the best when I feel like I have a project to do, and I'm connected to the people that I know can help me do it. I have all the resources I need, or I know where to look for them, instead of like continually coming up against delays. Obviously, you know, you can expect delays in life and every kind of project but when the delay hits, just come one after another is when you just really start to feel burnt out when you're trying to get something done, I think.

244

00:40:06.560 --> 00:40:14.389

Moderator: Alright. Well, thank you for all of that. We're gonna kind of transition into our last segment, Which is what recommendations do you have to improve Wellbeing at the school.

246

00:40:26.460 --> 00:40:35.139

I.1.2: the first thing I- I would like to say is like, I think that we both mentioned that and many people. We say that also, like we just want everyone to be like flexible but flexible, but accountable too. Like- and let it be flexible for the student, and also have the student find a way to be accountable for him or herself.

249

00:40:52.550 --> 00:41:01.090

I.1.2: And I realize that many students that are very happy with their PI I’m with a PI, who are very flexible. and also- and- they are- they understand that flexibility doesn’t mean like, just lay out, and then you don’t do anything, right? We need maybe some time to think about something personally. You have that time, but you can use also other time. Oh, yeah. personal time to be positive and always share What you need, even if it's just little things so flexibility is one thing. Another thing is like for resources we can have, like

254

00:41:36.350 --> 00:41:44.300

I.1.2: in large board like that says Okay, when you need this and even and call call those resources like have the postdoc call out what they need, because sometime we have like resources that are generally for everyone. But those are not what postdocs need. a postdoc- If a postdoc can call out what they need as a resource, and if that can be printed out and posted in their office: maybe name,and the phone number to call.

259

00:42:07.710 --> 00:42:13.960

I.1.2: That would be helpful, because finally, when I was able to get the name and the phone number to call the IT, it was so helpful for me. Now I will not wait like I used to wait.

262

00:42:21.440 --> 00:42:40.829

I.1.2: And also there's not just like IT, even like I can remember one day, also, I didn't mention that I was like in the elevator, and I was blocked in there, and I didn't know who to call, and I look at her in the elevator. I didn't see any number like the numbers I saw there was like. you know how you can calling over somebody from. another space, not at [blind] and help you. and then only when I managed to get out of the elevator, and then I went to a navigator, and she gave me a number.

00:43:00.460 --> 00:43:03.480

I.1.2: I feel very, very safe and comfortable.so I just use that number still there, that in the office, like some someone else can use that number right now. So have numbers like that, and the name of people to call.

00:43:16.450 --> 00:43:24.040

I.1.2: And the other thing also is really like sick and look at people. For instance, I.1.1 has a baby, and if she can park in school that he'll be so helpful for her.

00:43:28.680 --> 00:43:33.489

I.1.2: like for me like I have this Child that goes to- Goes to school.

273

00:43:37.980 🡪 00:43:39.120

I.1.2: And I feel like each time they may call me from the school. It was not possible for me to go to to his school and come back in less than 30 min.

275

00:43:48.180 --> 00:43:58.809

I.1.2: Something- something that can be done. If I had my car like close to available day at school. just like find a way to accommodate parents, for parents. That would be helpful, because sometime during my- my- my time like at lunch I could go back for

280

00:44:14.140 --> 00:44:19.869

I.1.2: 30 min, Max. 40 min. But that will not happen if I have to to take a bus. Just like find a way to accommodate parents.

283

00:44:26.440 --> 00:44:28.250

I.1.2: But again, again..

284

00:44:29.510 --> 00:44:33.420

I.1.1: yeah, I wanna reiterate that for sure, I think the resources for parents could definitely be improved, particularly parents who are postdocs. I think, because I was able to find some connections through like [blind] employees and support, for, like daycare or resources like that that I found on my own through the school, like I had no idea who to talk to, to try to find those things. All of them were still completely unaffordable as a postdoc like on our postdoc salary, though, like none of them, would have worked for me, cause they're still far too expensive. But I think I just like that was the probably the single, most biggest source of stress through the whole year was trying to navigate the program as a parent, and some of the things like I.1.2 was saying. Like, you know, something's wrong with my son. I can't just get to my car and get home in time, and like an hour and a half, because I have to wait for a bus, and the buses don't come all that often. They might be full, that you know all of those different things. and also just like I would have been helpful for me to have, like a point person to discuss, like what my resources were without having to feel like I needed to approach my PI about it. Cause. That's kind of an awkward conversation to have to talk to them about like- like I was saying some of the challenges with being a nursing mother like I need a refrigerator. I need you know, to to keep supplies, and I need access to lactation rooms, which are sometimes, you know, they're they are available. But these are things that, like I had to figure out on my own and figure out how how to make it work around all the meetings that we're expected to attend, and I don't know when I'm allowed to step out because I have to, or if I'm expected to just deal with the expectations and like I just didn't feel very supported as a parent, and some of that is, maybe I could have been more proactive to seek things out, but I didn't know who to talk to, so.

296

00:46:37.400 --> 00:46:40.249

I.1.1: I think that's something that could be improved for sure.

297

00:46:42.270 --> 00:46:57.520

I.1.1: I think that's probably the biggest one. I'm not sure that much else stands out to me honestly, because a lot of our resources that we have available within the program are are wonderful, and they're very helpful with, you know, supporting our our progress as as fellows. Maybe one thing, though, could have been emphasizing more heavily that, like

00:47:02.390 --> 00:47:08.239

I.1.1: our program, what's her position? What's Julie's position? is She a program like coordinator?

00:47:08.380 --> 00:47:10.690

I.1.1: or something?

I.1.2: I think so.

00:47:11.000 --> 00:47:16.299

I.1.1: It's just kind of like emphasizing that she can be your point person for questions like.

00:47:16.520 --> 00:47:38.139

I.1.1: if you don't know who to talk to email her. She can direct you in the right place, like if I had known that sooner, that would’ve helped a lot.

I.1.2: She helped a lot with like, all those resources. Like, she does say: “Here’s the phone number, here’s the name.” And that was very, very helpful.

00:47:38.220 --> 00:47:39.460

I.1.1: absolutely.

00:47:39.540 --> 00:47:41.759

I.1.1: I think one thing that like as a postdoc, some of those things, you know, once you make the connection with the person, it makes everything easier. But you're also trying to make sure that you're making a good impression on these people. And so I think it's important that the school itself is presenting the resources they have available like, I don't think we should always have to go searching for who can help us. Because we're also worried about making a good impression on our mentors and the people that we're working with. And

00:48:16.370 --> 00:48:25.690

I.1.1: yeah, that sometimes there's like little bit differential, like power dynamics through some of the people we might need to be asking for support from. And so I think it helps when you know the- our options are made a little bit more

00:48:31.820 --> 00:48:46.669

I.1.1: apparent to us from the beginning. Again, especially as parents, but in in any type of way that you might need support as a postdoc everyone has the different ways that they need to be supported. This just happens to be mine.

00:48:48.860 --> 00:48:59.009

Moderator: for sure. I do kind of wanna circle back and elevate one of the points that you brought up, which is the idea of having a person who could support you for kind of more of those personal needs like you said just simple things like having a fridge that you have access to that you don't have to like. Ask a supervisor about. I do kind of want to know what role you imagine this person being in. Is this a person who's kind of one of the other faculty members, or maybe an older year, or just someone kind of completely outside of that academic network, That's kind of just a support person?

00:49:36.230 --> 00:49:37.350

I.1.1: I guess. I guess it could go a couple of different ways. I kind of envisioned that it would be helpful to have some kind of like parent services person, for anyone at UNC. Like students would have some of these same challenges, too, and I- So I don't think it needs to be specific to the fellowship or even to the pharmacy school.

00:50:01.880 --> 00:50:24.470

I.1.1: And maybe there are things like that I just never knew about. But it seems worth it that someone from you know, UNC could hold that position to like have like a centralized ability to help anyone who needs support as a parent who's like connected with with UNC in any capacity. But I also think that

00:50:26.240 --> 00:50:29.639

I.1.1: you know, if it were within the program.. I- I mean I quite frankly, I wouldn't have felt very comfortable if it was coming from like my PI, or it's like the director of the program, or anyone who's like in a position of authority over like my work and who I'm reporting to. II think that that wouldn't be an ideal situation. I wouldn't feel that I could be candid with them about my needs.

00:50:54.910 --> 00:51:01.319

I.1.1: So I do think it would be better if it was someone a little bit more removed from anyone like I reported to. If that makes sense.

00:51:02.860 --> 00:51:03.910

Moderator: Okay.

00:51:04.020 --> 00:51:10.070

Moderator: thinking about sort of the the scope of this study and that we're- we're a part of the school pharmacy trying to look for recommendations. Do you think that there's anything that the school pharmacy in particular might be able to do?

00:51:23.290 --> 00:51:24.130

I.1.1: Hmm!

00:51:26.870 --> 00:51:29.080

I.1.1: I don't know. I mean, I guess- there could centrally within, like the school of [blind]. What do you call them?

00:51:37.630 --> 00:51:48.019

I.1.1: like student services, or like something like that? I feel like they could be a good resource for post docs as well, or like fellows, Cause, They obviously are primarily responsible for pharmacy students. But, we're kind of housed within the same building with some of the same resources and faculty. I mean, maybe that could have been a place for us to turn without feeling like-like directly- I don't know like I was saying, like we're directly reporting to those people in terms of like our job. There still could be some, I mean, maybe there's room for that within, like the graduate program as well. But I'm I'm maybe just not that familiar with it.

00:52:27.350 --> 00:52:34.210

I.1.1: But I would recommend, like, definitely within school [blind] or the program as a whole, just maybe centralizing some of the resources that are available for parents and like housing that within some kind of like student services support program-which I don't know if that's exactly right, cause we're not students.

00:52:47.270 --> 00:52:53.629

I.1.1: so I don't know if that would actually even be the right like department. But I would have found that helpful.

00:52:54.140 --> 00:52:57.579

I.1.2: Yeah, I don't know. Oh, Sorry I.1.1.

00:52:57.600 --> 00:52:58.950

I.1.1: No, no, look! Go ahead.

00:52:59.080 --> 00:53:05.189

I.1.2: you know. II just wanted to add, I don't know whether those resources that faculty do use?

00:53:05.210 --> 00:53:09.779

I.1.2: Maybe if I could say at the school of Pharmacy

00:53:10.670 --> 00:53:23.370

I.1.2: are they using? Do they share the same resources as the student? Maybe those can be also shared. For instance, I don't know how the nursing home look like. I don't know if they see hmm.. Possibility to add more nursing homes.

00:53:29.390 --> 00:53:30.459

I.1.1: What do you mean?

00:53:30.780 --> 00:53:32.939

I.1.2: like, will you-

I.1.1: I didn't hear you.

00:53:33.710 --> 00:53:39.889

I.1.2: for instance, in the nursing. How do you call that place where you need to go and pump?

I.1.1: Oh, oh, oh. Lactation room?

I.1.2: Oh, lactation room!

00:53:40.840 --> 00:53:53.119

I.1.2: so I don't know. I don't know if you can add more resources, for- somebody can add more resources for for nursing, not just like Postdocs, but for like student also, and also faculty.

00:54:02.310 --> 00:54:16.910

I.1.1: Yeah, I could. That would have been helpful to my knowledge. There's only one lactation room in the School of Pharmacy. And I mean, there's probably periods of time where that's enough. But there was definitely other nursing mothers on

00:54:17.130 --> 00:54:35.580

I.1.1: on campus that whenever I was there, so it was frequently full when I needed it. You know, everyone has breaks at the same times, and you're probably gonna need it for half an hour at a time. So if there had been more, that would have been great, because my alternative was like having to walk over to the hospital, which then adds another however much time to my day, that's taking away from everything. So that's a great suggestion, maybe more- like a refrigerator that could have been used, or another lactation room somewhere that could have been used.

00:54:51.130 --> 00:54:57.200

I.1.1: I would have been able to use a private office, but I don't have an office, like I share an office, so I couldn't have used that

00:54:57.340 --> 00:55:00.760

I.1.1: That's a good point as well.

00:55:03.390 --> 00:55:07.590

Moderator: Alright, since we're getting a little bit closer to the end.

00:55:07.660 --> 00:55:18.129

Moderator: I do kind of have a wrap up question. What other thoughts or suggestions would you like to share that you feel would be important for this work?

00:55:27.330 --> 00:55:32.339

I.1.1: I feel like we've covered quite a lot.

00:55:32.400 --> 00:55:35.840

I.1.1: Maybe the only thing we haven't covered too much is encouraging- encouraging people to allow their their post docs to work remotely a couple of days a week. I think it makes a huge, huge difference. II know some PI’s always allow that; some never do. But if you're not actually doing any lab-based work, it seems like a very reasonable request to make, in my opinion, and it really promotes, like a much better work-life balance, and like sense of well-being when you are able to avoid some of those major stresses of commuting that we've talked about. So that would maybe be my last thing is just creating a culture where that's a little bit more acceptable widely among the faculty and the mentors.

00:56:30.730 --> 00:56:41.249

I.1.2: Yeah, and yes, and also this something called the wellbeing days at school, and as postdocs, We didn't know if we are sharing those days or not. So maybe like officially extend that to all the postdocs, too.

00:56:48.760 --> 00:56:52.010

I.1.1: Yeah, yeah, they, I was told that didn't apply to us. So that would have been nice if it did

00:57:01.370 --> 00:57:07.040

Moderator: alright. So that concludes our discussion prompts for today.

00:57:07.070 --> 00:57:24.610

Moderator: If you have more that you would like to share, either just after this meeting or later on, that you have something that comes to you. We do have a post survey link that was in

00:57:24.660 --> 00:57:30.920

Moderator: the calendar Invite, and I'll go ahead and grab that link as well in just a moment.

00:57:31.020 --> 00:57:32.790

Moderator: So that

00:57:32.800 --> 00:57:44.180

Moderator: it's in the chat, and you can add any responses that you might want to include, that you didn't have time or just didn't think of today.

376

00:57:45.380 --> 00:57:48.319

Moderator: So I will go ahead and put that in the chat as well. It's also on that outlook calendar invite that you got to come today. So it's also there, save for you. If you come back like tomorrow and realize there was something else you wanted to share.

00:58:03.100 --> 00:58:26.000

Moderator: So you can anonymously provide any additional feedback or perspective not shared during our discussion today. So thank you very much for participating in this focus group. Your input is obviously very valuable in helping to inform and support future well-being efforts. So thank you so much. And that wraps up our discussion for today.

00:58:26.440 --> 00:58:29.339

I.1.2: Yeah, thank you. Appreciate your time.

00:58:30.520 --> 00:58:31.060

I.1.1: Good luck.
