# Supplementary material for: Assessing factors that influence perceived burnout in postdoctoral fellows and identifying recommendations to support their well-being
Source: PLoS One. 2026 Mar 17;21(3):e0344974. doi: 10.1371/journal.pone.0344974 (PMC12994809; doi:10.1371/journal.pone.0344974)
Supplement: S3 File — (DOCX) [file pone.0344974.s004.docx]

00:04:01.150 --> 00:04:02.989

Moderator: Hi, A.2.1, how are you?

00:04:04.800 --> 00:04:07.790

A.2.1: Hey? Moderator, how are you? And it's prono[blind]ed “A.2.1.”

00:04:08.030 --> 00:04:09.370

Moderator: okay, “A.2.1”?

A.2.1: Yeah.

Moderator: Okay.

00:04:09.480 --> 00:04:15.550

A.2.1: Let's see if we can get my video. Okay, yeah, there I am.

00:04:15.640 --> 00:04:16.700

Moderator: cool. And then I think we have 2 other people joining us. So we'll just get started when they join.

A.2.1: Okay, Sounds good.

00:04:52.650 --> 00:05:09.350

Moderator: Alright. I think we have everyone here now. So I'll go ahead and get started. and I do have sort of a semi-structured script that I'm going off of. So if it looks like I'm looking away from the camera, it's just to keep myself on track.

00:05:09.940 --> 00:05:16.010

Moderator: So my name is Moderator. I'm the Moderator for today's focus group discussion. During today's focus group, we'll discuss factors which influence your well-being as a postdoctoral fellow at the school as well as your recommendations to improve your well-being. So I am a PharmD candidate here at [BLIND]. And this is the research project that I'm working on. So if you feel comfortable we'll go around the room and just kind of introduce yourself: Who you are, which division you're with, that kind of thing.

00:05:54.500 --> 00:05:59.990

A.2.1: I'll go first. My name is A.2.1. I'm a postdoc from [Division].

00:06:11.230 --> 00:06:12.610

A.2.2: can I go next?

00:06:12.930 --> 00:06:13.660

Moderator: Yeah.

00:06:14.270 --> 00:06:22.000

A.2.2: Yeah. So my name is A.2.2, and I'm a postdoc associated with the school of [[blind]]. And I'm working on the [blind] program.

00:06:24.630 --> 00:06:25.550

Moderator: Okay.

00:06:37.280 --> 00:06:39.220

A.2.3: Hmm.

00:06:40.850 --> 00:06:41.949

A.2.3: can you hear me?

Moderator: Yes.

00:06:42.150 --> 00:06:47.890

A.2.3: Hi, yeah. My name is A.2.3. I'm a postdoc with the [[blind]] program here at [BLIND] School of [[blind]].

00:06:53.260 --> 00:06:54.980

Moderator: Okay, wonderful.

00:06:57.200 --> 00:07:07.710

Moderator: So, you received a copy of the informed consent when signing up, so I'll just briefly go over that information now. This focus group is being recorded so that we can obtain accurate information about what was said. All data collected will be kept confidential. We ask that you keep the discussion confidential as well for the privacy of your peers.
 Your participation in this focus group is entirely voluntary, and you may stop participating at any time. You do not need to answer any questions you don't wish to answer. And as I ask questions, feel free to respond not only to my questions, but also to others responses During the discussion.

00:07:44.970 --> 00:07:51.440

Moderator: Before we get started, I would like to clarify a few terms for the purpose of the discussion today. When I say, wellbeing, I mean overall judging life positively and having an overall state of contentment, and when I say, burnout, I am referring to feeling mentally exhausted, and having negative emotions about yourself or others, and decreased motivation and perceived performance.

00:08:12.610 --> 00:08:27.650

Moderator: So the school has begun assessing wellbeing amongst- among its community members, including faculty, staff, students, and postdocs. Findings from these assessments have helped inform strategies to improve wellbeing at the school. And as an extension of these assessments, this focus group study aims to identify school-based factors influencing postdoctoral fellow wellbeing and burnout and identify recommendations to improve well-being.

00:08:44.260 --> 00:08:44.920

A.2.3: Hmm.

00:08:45.270 --> 00:08:56.069

Moderator: So with all of that said, we'll go ahead and jump into the first question, what factors do you feel positively impact your well-being and bring you fulfillment?

00:09:00.420 --> 00:09:01.430

A.2.3: Hmm.

00:09:03.130 --> 00:09:10.529

A.2.3: I guess for me, stuff working in the lab always helps my wellbeing (laughs), you know. I mean, it's failure's a part of science. But having some wins always, you need some wins. Can't be all negative all the time. I think that was- that definitely helps my mood.

Moderator: Mm-Hmm.

00:09:27.140 --> 00:09:36.899

A.2.1: Does it have to be like within the school? Because I don't know if anything necessarily influences my well being directly at the school level right now.

00:09:42.350 --> 00:09:43.400

Moderator: Okay.

00:09:43.920 --> 00:10:01.430

Moderator: Umm.. Some specific probes that we might ask about could be your relationship with peers or your supervisor at the school; workload, work-life balance or integration. Those could be some specific things.

00:10:01.690 --> 00:10:08.880

Moderator: Or if your well-being you feel is not really related at all to the school. That's also (nervous laughter) an acceptable answer.

00:10:09.000 --> 00:10:20.870

A.2.1: Yeah, I don't think I- I guess, am I? There is kind of flexibility that I do have in my postdoc, that I probably wouldn't have anywhere else. So I think that flexibility affords me- You know, more time to be at home, which is where I think I kind of get my positive wellbeing and things like that. So I guess from the school perspective more so flexibility.

00:10:45.310 --> 00:10:48.050

A.2.3: I think I like the- the health insurance plan is decent for postdocs, like, it's better than- I've worked in industry before, and this is probably one of the better plans I've had. Like I met my deductible and so like it's covered! It’s something rare. And so that makes me feel well. Like in the- to follow on A.2.1’s point like the flexibility like, if I need to go to the doctor, it's not like a big Kerfuffle, to- to go. I just go to the doctor. So like, “I'm stepping out. I'm going to an appointment.” Stuff like that. So I think the flexibility to take care of life events is nice in the- in- in an academic environment.

00:11:35.450 --> 00:11:45.710

A.2.2: But I would say that flexibility at the work schedule like it's like 9 to 5 job. But sometime, you know, you don't have experiment, and you can come early. And when you have experiment to go and do experiment, so I have heard many, many postdoc labs. They don't have such flexibility. But in my lab, luckily I have that. So I think that is a kind of positive environment. And I feel like going everyday to lab to work hard. So, yeah, that’s what I say.

00:12:06.370 --> 00:12:08.600

A.2.3: yeah, on the flip side, like. There are times where you just have to be there, like, you know, like, I- I do animal work. Where like, stuff is you- You go when the animals are ready, and if it turns out to be a long day it will be a long day, so like there are days we don't have it, but for the most part, you can kind of control your schedule, at least at the postdoc level more so. It's both up level.

00:12:37.410 --> 00:12:51.320

A.2.3: But I will say, one stressor for me is, you know, in the postdoc, like you're trying to navigate your career goals with your PI’s like goals and so trying to make sure you're getting what you need from the experience is always like a source of frustration for me, because they may not exactly align. Because they are trying- You know, I'm not 100% sure if I want to do academia. But I do want it to be a worthwhile experience, and like the things I need may not necessarily fit with what their, like, Their grant structure, or something like that. So I, that is one source of stress for me is like, make sure my career is progressing in a manner that I think is beneficial to me. which may not necessarily align with what the funding mechanisms that pays my salary and stuff like that.

A.2.2: Sure.

79

00:13:41.800 --> 00:13:42.950

A.2.3: That's- Also money the money for-

A.2.1: Uh, like, that’s like the number one stressor! (Laughs)

A.2.2: I think that is a negative effect, not a positive one!

00:14:04.540 🡪 00:14:09.570

A.2.1: I- we don’t- I literally just had a conversation with my chair earlier today. Because I applied for a K and I got a pretty good score for the first round and resubmitted.

00:14:14.070 --> 00:14:31.350

A.2.1: and so like I'm a fifth year postdoc, so like nobody wants to be a postdoc forever. So we were having a discussion, and she was like, Well, you know, we're just waiting to hear back from the K. Before we move forward, and I told her I said, Well, if there's no budging on the salary, I don't even want the K. Because, like what's- what's the benefit for me if it means another 2 years of making money that you know, I barely can keep myself afloat. So the money is definitely a stressor, especially considering the work that we do. I feel like I do a lot of good work, and I put out a lot of things that some of the faculty and staff aren't even doing. And so it kind of sometimes to me, feels like a slap in the face. And I'm doing all this work, putting all this effort in. And I get like half of what- Not even half of what some of these faculty members make. So

00:15:11.760 --> 00:15:18.970

A.2.1: that definitely is disappointing, and contributes to a negative wellbeing.

00:15:20.120 --> 00:15:42.069

A.2.2: And we can't even negotiate for salary in Academia. That's a main drawback, and I'm surprised that in many universities, the postdoc salary has been raised. For example, at [[blind]] or [[blind]], but in [BLIND] they haven't increased it a bit, so I was bit surprised and kind of irritated also, at the same time.

00:15:45.710 --> 00:15:52.840

Moderator: Gotcha. Well, that's definitely good to know. I think that was brought up in one of our other focus groups actually. Are there any other factors that you would like to talk about as far as positively affecting your wellbeing?

00:16:03.620 --> 00:16:07.800

A.2.1: I feel like early. I feel like how I probably felt about my postdoc, when I first got here versus now, are 2 totally different experiences, so like when I think about initially when I first got here. Then I- I would say, like. “Oh,” like you know, “My co-fellows were contributing to that cause.” I was like we- would go out frequently, and things like that. But then it kind of, I think now I'm at a place where I've seen so many fellows and postdocs, like, come and leave that. It's hard to really even establish, like a relationship with some of the new ones, because now, like they're so much younger than me. And so what used to be like, oh, I can go to my co-fellow, and we have similar experiences, It’s not necessarily the case now, like I’m I’m- at a point where some of the PharmD students that I had like when I first started my post are now becoming postdocs, and that even feels weird. So

00:17:04.650 --> 00:17:16.389

A.2.1: I would say, like initially, maybe, like, for a traditional person who does like maybe a 2 year post doc. Then their peers, and, you know, just like, get coming out- like for me, I was coming straight out of my PhD program. So anything is better than you know, having to figure out how to do your dissertation, all of that stuff. So you're having fun. But now, as a fifth year, I'm kind of like a little jaded. And that's like I don't have those type of positive- I think you could say experiences that I probably had the first 2 years as a postdoc.

99

00:17:41.490 --> 00:17:42.490

A.2.3: I think-

Moderator: Yeah-

00:17:43.500 --> 00:17:52.340

A.2.3: Go ahead.

Moderator: Go ahead.

A.2.3: Yeah. I- I think [BLIND] does a pretty decent job of onboarding to like II just started my post, Doc, in June and so they do a good job of telling you the different resources, and like telling you different grants you can apply for and stuff like that. So they they do make you feel.. I don't know if it's the best thing I maybe it's they they give you rose colored glasses to like your state of affairs in terms of your chances of making it, which I don't know if it's a good thing or bad thing, but they do try to make it seem like, Oh, yeah, it's- it's uh reasonable you're doing a postdoc.

00:18:25.100 --> 00:18:31.180

A.2.3: But yeah, like, I can imagine when you actually need to leave. I don't know if there is welcoming and like- or if they or they have the same support. But initially, when you're just starting, I feel like you're well supported.

00:18:43.990 --> 00:18:47.539

Moderator: A.2.2, where do you stand on those issues?

00:18:49.120 🡪 00:18:57.830

A.2.2: Yeah, it was good. I agree with A.2.3 that’it's very supported. So yeah, th’re's no doubt about that.

00:18:58.310 --> 00:19:10.060

A.2.2: They they give information about everything for the international scholars and everything. Yeah. But I have a negative prospect of- regarding the visa immigration issues.

00:19:10.210 --> 00:19:40.309

A.2.2: because I am from [blind], and we have a like 2 year of home residency rule after completing the 5 years. So because of that, we need to apply for the waiver to switch to other kind of visa. So most of the professors, I think they are not knowledgeable about that procedure, and also the HR. And it's kind of make me crazy to make them understand why it is so important why, the timelines are very important, because there shouldn't be any gap when you are transitioning from one visa status to another, because you will be losing your legal status within the US. That would would be wrong. So I think, that

00:19:53.200 --> 00:20:18.740

A.2.2: is the one thing that the mentors of the pi. They should be aware of these factors. And also there might be some educational program regarding those before hiring any international student who has a J one visa rule. So if the pi knows those. So that would be very helpful when the international student comes here and also same goes with the HR. Also.

00:20:18.960 --> 00:20:42.380

A.2.2: because sometime they said, Okay, we will do it in the when the time comes. But the thing is, these things takes a long time. For example, the waiver is takes longer time. So we have to be on a tight schedule of applying and everything. But they took it very casually. So you have to keep pushing them, pushing them to which is not good. So that is a kind of negative effect. Yeah.

114

00:20:42.890 --> 00:20:46.359

A.2.1: yeah, can you remind me how to pronounce your name

00:20:46.960 --> 00:20:57.190

A.2.1: is it (attempts pronunciation)

A.2.2: “A.2.2”

A.2.1: Okay, I just wanted to kind of echo with what A.2.2 said. So I'm not a international student, but I think something that I think is lacking- That contributes to negative- I like, I don't know. I feel like we're talking all about the negative things, but negative wellbeing is when she said that it made me think about DEI, and how they talk a lot about, you know, wanting to recruit and/or retain, like diverse students or postdocs. But I don't know if the system as a whole is set up to even provide the resources in order to do what they have set out to do. And so, if that means for A.2.2, like, you know where are the resources that she goes to make sure that her, you know, visa isn't lapsed or anything, or thinking about me like my focus is in rural and Black communities like trying to figure out, who do I go to to kind of like do the type of work that I wanna do. And so I think that there's kind of a lack of of knowledge for resources that you know, bringing in diverse talent, kind of needs, a certain type of learning that the faculty members are kind of like, not. They don't have any knowledge, or HR People like that.

00:22:12.350 --> 00:22:23.369

Moderator: So I guess to try to paraphrase what you're saying. There's a lot of talk about DEI, but maybe not a lot of the infrastructure and support that's needed for it.

A.2.1: Exactly. Exactly.

00:22:29.120 --> 00:22:42.380

Moderator: Alright. Well, we've kind of already transitioned into it a little bit, but we'll go ahead and formally transition to- into any factors that sort of negatively impact your wellbeing or contribute to your burnout.

00:22:44.260 --> 00:22:49.460

A.2.1: I would definitely say the lack of diversity for me is kind of draining. It seems, I think, for some people it seems like something that's overlooked, but I- I feel it on a daily basis. I'm not sure how A.2.3 and A.2.2 feels but I know, like within- I feel- I feel like my division is a little more progressive when it comes to a lot of things that I do feel comfortable around them. But when I look at the University as a whole, I feel out of place. And so

00:23:21.820 --> 00:23:40.640

A.2.1: I think it's sometimes, you know I that's why I do feel more comfortable at home or with family, because, you know, no one wants to feel like a outsider, you know, at the place that they work and things like that. So I think that that does kind of contribute- contribute negatively to my experience.

00:23:44.240 --> 00:23:46.140

A.2.3: Yeah, I agree with that, like, you know, the- you know, the- and I- I feel this is done everywhere. But like. yeah, they'll, you know, for photos, you know, They'll make sure they'll have a mosaic of you know pretty much every facet of you know society in- in like their website pages. But like, if you actually go to like rosters and stuff like that, you know, I did my graduate studies here at the [blind] Department. I think there was like, maybe like two-myself and one other person-that were people person of color.

134

00:24:23.400 --> 00:24:26.790

A.2.3: And you know, in school [blind] I don't. I don't. You know. If you're you're few and far between. And I and I I've come to accept that somewhat which is not not a great attitude, but like I kinda just accept that. But I guess one thing I am concerned about is the- the whole thing with the 1619 project the [blind], and whether she was going to join the school of journalism, and they offer her a position, and the [University board] rejected it. And so it's like. You know, someone- If I was entertaining going like an academic route, having, you know- what if I work hard and like I earn a spot just to have it taken away, because it's like some political thing. I don't think

139

00:25:11.170 --> 00:25:13.600

A.2.3: the University ever really dissuaded me of that notion that like that's not a possibility here, or, like, you know, properly communicated, and like not to say I, am in the running for any sort of tenure track position. But if I was..

142

00:25:28.180 --> 00:25:38.919

A.2.3: that's like concerning for me, and like I- I have that in the back of my mind that, like oh, I could just easily be a political pawn, because, like, regardless of my academic work because of my- how I identify demographically, they'll think I'm just placeholder hire and like, and you know, like like you know, quote unquote “woke,” I don’t know, but like it's clear that there's a political element to how the university is governed, and that that's very concerning to me, and I don't think the- the university has communicated

146

00:26:02.470 --> 00:26:18.249

A.2.3: how they're gonna push back to that. At least they there might be-

A.2.1: They DID communicate! They’re not pushing back.

A.2.3: You could just say, they're just not gonna do anything. And then that that's all. I need to know that, like, regardless of what I do here, there's no future for me here. you know, and that's a definite negative to my wellbeing is knowing that, like, you know outside, regardless where I do, what you're going to see is my skin color, or my demographic, or like, I'm not a US citizen or something along those lines. You're gonna see that. And that's gonna steer, whether you're gonna be, have actual, have a shot at something versus your your performance. And so that I- I- I do feel like that the university does a poor job of just, you know, of showcasing otherwise after the incident.

00:26:59.240 --> 00:27:11.789

A.2.1: Well, you- in August, was it in August? No. may- Maybe it was around this time [BLIND] did come out in support of, like the Supreme Court's decision for free speech and our compel speech, I think it is. And so it’s their way of saying like, they're not gonna necessarily hold it against you, but like people that I guess might be against you like they are also welcome to speak. So then it becomes an issue of again, Where's the support? You know? I guess. So.

00:27:35.960 --> 00:27:40.170

A.2.1: Yeah, that that is something that has bothered me because I am interested in academia, specifically a tenure track position. And you know I do have concerns on the daily of, you know, thinking about how [BLIND] is run by the [University board], and how closely that is tied to politics, and those politicians that any day could say, “well, we don't like the work that you're doing, and so we don't see any value in it.” And so, you know.

00:28:07.130 --> 00:28:14.970

A.2.1: they could just be, do away with me, and so I- that's why I think also that fully contributes to again the jadedness I have like when I first started [BLIND]. And then, like, I started like Pre Covid, like everything, was all happy-go-lucky, everybody was talking about DEI, and then, you know, to see how things have progressed through Covid. Now, even with you know

00:28:31.820 🡪 00:28:41.389

A.2.1: I- I want- I- I hate to say, like, but the shootings like feeling unsafe on the campus, you know, and things like that. So it's it's very hard.

00:28:44.310 --> 00:28:45.219

Moderator: For sure.

00:28:51.430 --> 00:28:57.750

A.2.3: Yeah, like, I think. Like, you know, my work isn't inherently, like, political, right? With this pancreatic cancer like there's no, there's no real- there's no politics in cancer. Well, in cancer, it shouldn't be. I mean, you're sick, You're sick.

00:29:09.130 --> 00:29:19.359

A.2.3: so like. On the one hand, I feel that kind of dampens that, like if I was doing something that was edgy or controversial, inherently or like, you know, counter- you know, ruffling feathers rocking the boat, so to speak. I’d feel- I definitely wouldn't even be here, I think, but even like something as- should be above all, the all that, like cancer researchers, you know, in like school of [[blind]] and stuff like that. I do worry about the undercurrent of like. Okay.

00:29:42.200 --> 00:29:46.250

A.2.3: you know, you have to be exemplary to- to offset the notion that you're just a- to counteract the argument. Why- “Why are you hiring this person? Are you just doing it to be like- you know, you know, just to to have a minority on your in this department, or something?” because, like school of [[blind]]’s faculty is very homogeneous like it’s- it's not.

00:30:12.840 --> 00:30:18.060

A.2.3: There's not much diversity there. And so like, you know. So you you have to go above beyond to showcase that you're not that token. And I think that's again not helpful to your well being to that you have to deal with that.

00:30:35.490 --> 00:30:36.589

A.2.3: (to A.2.2) You're on mute. You’re still on mute.

00:30:42.120 --> 00:30:51.769

A.2.2: Yeah, yeah. Sorry to. Yeah. So.. sorry. I have to leave early from the meeting. But before I leave there are 3 points that negatively affect me I would like to mention. First was the salary structure. I'm not happy with that. I'm saying it openly. And number 2 thing is the visa thing. the transition could have been very smooth, but because of the lack of knowledge, it’s not smooth. and it gave a lot of stress and unnecessary headache. regarding the fact that we have to work hard in the lab.

00:31:21.610 --> 00:31:25.339

A.2.2: and number 3. The third factor is, what's the limit of working hard for a postdoc? Because it's expected the postdoc will work really hard. But I don't believe that, because we are kind of done, we have done. PhD, we are already doctorate, and we are on the higher level, I believe. So why we have to work so hard, better than the PhD students? It should be in the reverse, because, PhD student, of course, they should work hard to get their degree. But why the postdoc have to do such kind of work to get Len- to know. I mean by the PI to get the recognition. Everything, even if you work hard, the PI is never satisfied. they will say, “Okay, I expected a bit more.” So what's the limitation of that? I don't know. That's one. And the thing is also, when you work hard. You work in the collaborations.

188

00:32:16.300 --> 00:32:45.000

A.2.2: For example, for me past one year. I have also hard for 2 projects, and both papers are submitted but because of the collaboration thing, it got delayed, delayed, delayed, and the paper is not still published yet, which is really frustrating, because I also need to apply for the green card application, where I need the citations of my paper. So when like this paper are not published, and it's in the somewhere hanging. And I don't know what's going on. So that is also a kind of critical issue when you are working in a collaboration. So that is also negatively impacting me.

00:32:53.310 --> 00:32:55.620

A.2.2: yeah, because of the thing. Yeah.

00:32:55.780 --> 00:32:57.220

Moderator: thank you for that.

00:32:57.330 --> 00:33:24.040

Moderator: I'm gonna go ahead. You said you were leaving early. Right?

A.2.2: Yeah.

Moderator: Okay, I'm gonna go ahead and just put a Qualtrics survey link in the chat. It's also in your outlook calendar invite so that if anything else comes up that you think of that you want to share, especially recommendations for how the school can improve your wellbeing, you can go ahead and put that there. And it's completely anonymous. Okay?

00:33:26.180 --> 00:33:27.190

A.2.2: okay.

00:33:27.420 --> 00:33:34.480

A.2.2: thank you. Nice meeting you all. I'll see you sometimes. Yeah, yeah. Bye, have a nice evening.

00:33:36.270 --> 00:33:39.299

A.2.3: I agree with her. You have to like run much faster as a postdoc than you were as a grad student.

00:33:46.740 --> 00:33:49.890

Moderator: And do you feel like the- that expectation is kind of clear made, clearly made to you, or it's just kind of that's the culture, or?

00:34:00.080 --> 00:34:04.820

A.2.3: I think it's. you know. You know, if you want to like rise, you know, build a profile that can make you attractive for tenure track you.. It’s sort of implied like you have to be productive, right? Like, you know, as soon as you start, you gotta be productive in showcasing. And like. And it's it's a harder.. It's a it's a it's a finer needle of thread, because you have to stake out an area that has the- the depth that you could be your own and individual investigator, like an independent investigator and while being separate from what your PI's doing right? You don't want to be a carbon copy of your lab, because they'll say, Well, you're not original, so why should we? Why do you bother?

00:34:45.590 --> 00:35:01.830

A.2.3: And so like that balance there. And so that's where that friction I was describing in terms of dealing with a PI is like, you know, you need to be yourself, not the PI. But you also need to publish, and they're only going to give you money to publish what they want to publish. So like that conflict is there. And I think it's implied. Like, you know, I I'll probably you know- things don't work out, I'll go into industry,

where, you know. I'll just say I'm out of here. But

00:35:14.570 --> 00:35:20.349

A.2.3: the- you know, if you're trying to stay on the academic track you're like, beholden to your supervisors. And so that’s implied, like, I know, I got to please them, all right, cause if I apply for any job I'm going to say, well, I need a recommendation letter.

00:35:29.120 --> 00:35:40.580

A.2.3: and, like, you know, it's not like industry recommendation letters where like, do they do their job? It's like pretty extensive like they like, Write like a narrative on who you are as a researcher. And so you really have to you know, I guess just kiss their ass almost like to make sure they have, like an you know, they can write a really really strong letter, but you may not necessarily jive with them entirely. So it's like that's. And so you kind of like. That's a point about wellbeing so like, I know this person has my future in their hand.

00:36:01.890 --> 00:36:07.509

A.2.3: and maybe you don't get along with them like I- Fortunately, I'm not in that that sort of situation. But you know what if we have a disagreement that we can't work around, and then it becomes adversarial. Then I know my career is over, right? Like essentially my career ends because. You know they’re that- they have that much weight and sway over your career and academic tree. So it's that that concerns me. I don't know if there's a way around it. I think it's just endemic of academia is that you have to just survive like that apprenticeship program unscathed and like a lot of people don't.

00:36:48.840 --> 00:36:58.139

Moderator: All right. Is there anything else that either of y'all wanted to bring up about factors that negatively contribute to your well-being or burnout?

00:37:02.130 --> 00:37:05.929

A.2.3: I think I covered all mine.

00:37:06.560 --> 00:37:10.110

A.2.1: Let's see. So so far, I think we talked about finances is being number one. Seems like lack of resources. I know, [blind] said about, you know, the visa.

00:37:21.760 --> 00:37:29.880

A.2.1: and what was the third one? She said. I just wanna make sure that we covered it. I think most of mine all come- like stem from the lack of finances.

00:37:30.630 --> 00:37:38.920

A.2.3: Yeah. Her third one was like the the expectation of like, you know, why am I still working at like at a fever pace.

00:37:39.340 --> 00:37:46.359

A.2.3: which II kind of tie into like you need to please your supervisor right? Like they set the expectation. And there's crazy people out there right? Like.

00:37:49.270 --> 00:37:59.739

A.2.3: you know, you think about- these people don't have any training. They're not like in industry, like when you become a manager. Usually you take a class to learn how to get like.. These guys just get a b[blind]h of money and told to hire people and some of them don't have personalities that they should never hire. They should never be managers of anything right?

A.2.1: You are *so* right.

A.2.3: They're, you know, narcissistic, egotistical. All this other stuff, so many personality flaws. And I- you get to an industry. But like here, their power’s like unchecked right? like even like, the you know..

00:38:22.530 --> 00:38:31.570

A.2.3: it's- you hear horror stories of like just psychopaths that have labs, and like, you know, if they have enough cash in at the right institution. there'll always be a sucker that will say, Oh, I want the name brand thing. And I'm going to. you know, power through. And they just get destroyed. Or they, you know, they come out really damaged. And so..

00:38:41.630 --> 00:38:53.750

A.2.3: Having proper training of these people who never worked= really, most of them never been anywhere else, so they don't know anything on how to conduct themselves just to be like an effective manager or leader. I I'm sure they're doing stuff, but they need to do more. Just cause yeah, that A.2.3ers, I think.

00:39:05.070 --> 00:39:18.870

A.2.3: I will say on the positive side, I know when school of [[blind]] was, I think they were trying to recruit or evaluate people. They did have a seminar, and they had invited students and postdocs to go sit at the seminar and actually have lunch with the with the candidate, and then they sent out a survey like.. In it, they actually asked for feedback so you could get a feel- so you it was nice to see that you were included, and maybe you could see like oh, I got. I don't know. I don't know how much weight it has in the overall hiring decision, but the fact that they asked, you feel like, oh, you know, maybe they do give some consideration to that.

00:39:42.650 --> 00:39:44.240

A.2.3: but

00:39:44.820 --> 00:39:45.500

A.2.3: yeah.

00:39:45.680 --> 00:39:53.950

Moderator: alright well, then with that, we'll go ahead, and move into the final section, which is what comes to mind as far as recommendations to the school to improve wellbeing?

00:40:06.090 --> 00:40:09.039

A.2.1: I think, something that A.2.3 mentioned about.. You know, if- I think there needs to be some type of class that people who have postdocs take like, you know, when I think about becoming a postdoc like I had to sit through so many trainings to make sure I did this; I knew where this was; I feel like the same should be- like the same responsibility should be held for the PI’s. Because like, I said, luckily I was in a division that kind of was able to help me navigate. But I've met so many post docs throughout my time here that they don't even know where to start, and then they go to their PI, and they're like, I don't know, you know, like. And so again, just going back to

00:40:50.800 --> 00:41:03.529

A.2.1: what A.2.3 said about a lot of these PI’s are just unchecked. And it's mostly like, okay, I just got funded to hire somebody to basically do my work. Cause that's really how a lot of people see postdocs like they're just cheap labor to do (laughs) whatever I need, you know, done for this research or whatever I feel. I feel like there should be some type of requirement for people who hire postdocs. And again thinking about, you know..

00:41:16.520 --> 00:41:43.700

A.2.1: some of the things that we've talked about that goes through like, well, finances. So like, what do you do? If a postdoc, you know, has issues financially like, do you know where to go find the resources? Or if there's a visa issue like who do- that, who do- Who does the PI reach out to? There’s a visa issue, you know. And then, like just some understanding of the power structure that I think we deal with on a daily basis cause..

00:41:43.940 --> 00:41:57.430

A.2.1: even though I- I have a great relationship with my PI like there is still kind of some reservation, because she's the chair of my department, too. So you know, it's like, even though I can communicate with her. You know, there, there is kind of like again. How- What's the check and balances process like, how can I really, you know, share how I feel about if I'm being overworked or being over extended, and things like that, and because, you know.

00:42:11.680 --> 00:42:19.550

A.2.1: We're at their mercy, you know, like you say, like for the next step. You know, it's- it's very hard. So I- I think there it is, just one of the downsides of being a postdoc, and being in this place in our career.

00:42:25.640 --> 00:42:31.449

A.2.1: But it is hard, I think, and I think it just fully just kind of hinders our well-being when you think about all of this.

00:42:40.000 --> 00:42:44.249

A.2.3: Yeah, I- I totally agree with that. I think maybe having like a like in your hiring contract, like some stipulation that you'll have something for your- your benefit. Like like as A.2.1 said, like, you know, they're hiring you as a grunt to crank out their work like. You're highly skilled and low, low cost person. So having. But like you also, you're- you're- you're- you're doing a postdoc, cause you are thinking about or not- have not closed the door to try and do academia. And so like, there are certain things you specifically need. And like when you look at a postdoc offering..

00:43:19.720 --> 00:43:24.750

A.2.3: Typically, they just list the project you'll be working on. But they don't really say like, well like, and you'll have this piece that *you* can have or like.. I understand you may not know it right off the bat, but like having some protected time, maybe for the postdoc, where they get a chance to grow *their* career objectives as opposed to just doing what the PI hired. I understand, like, you know, and PI should just have to accept that like when “I hire a postdoc, that'll be 75%, my project, 25%, their thing right?” So they actually have a chance to move- progress, their career. I think that would be really, really beneficial.

00:43:58.460 --> 00:44:05.600

A.2.3: And yeah, like, any sort of training on like how to handle stuff a little bit better would be- would be also useful to just have them more prepared to do it.

274

00:44:12.160 --> 00:44:18.830

A.2.3: Also just money, just- just-

A.2.1: Like, for real!

00:44:19.030 --> 00:44:27.809

A.2.3: I said, the medical benefits are great; the retirement benefits are God awful right? And so like you had- they give you absolutely nothing, and I don't think that's fair. We're already like low paid. But to say, like, you're making 0 progress towards any sort of retirement is- is nuts and like. and that's like,like, unacceptable, in my opinion.

00:44:50.820 --> 00:45:07.779

A.2.1: And you know, I think, that this is an issue systemic to postdocs across the country like I think I even read somewhere that how no one's even doing a post-doc anymore because they're- they're like, what is the benefit like to it? ‘Cause we've already struggled through graduate school making like just minimum payment. You know, minimum salary through the graduate program. But then to come to a postdoc like, were you doing work that is you know, very highly skilled, you know, you think about some of the PI's that are making triple sometimes quadruple your salary, you know, like you're doing the same type of work and things like that. And so and I- I think again, if you have a- a PI, that isn't thinking about your next steps like the postdoc just becomes a waiting period until you're able to get out. But

00:45:47.720 --> 00:45:57.830

A.2.1: it it could be a like- There could be like no growth during that time period. It's just literally someone paying you to do their work until you kind of figure things out. And unfortunately, a lot of people have kind of I think, met that end to where, like their postdoc was just no longer serving, and they just found another job. And they moved on. It seems like there's, you know, at the end of your graduate program like you work hard. But you know you're gonna get your degree. There is no real incentive for your postdoc. I feel like, even when it comes to like whether or not the University will retain you as a faculty member. Even like, you know, research science is, there's no guarantee for that. So I feel like thinking about ways to improve.. It is, you know, creating some type of incentive for people to want to do postdocs, whether it's a, you know, direct track to a- a tenure track position or a direct pipeline to, you know, some other type of research institution. Like, if it's someone wanted to go to RTI or so, you know, like some type of pipeline that progresses the post doc versus, just, you know, waste time until they find something else.

00:47:05.580 --> 00:47:09.580

A.2.3: Yeah, that's a good point, I think, like, uh, career services could do more on that. And like, maybe, like. Tell you where you stand like, maybe the school has to give you: So um, like, listen, you have like a 0% shot, like, you know, like of being at least put me out of my misery here rather than have me waste my time. I think that would be helpful like, because, like the career services- I think at the graduate level, it really doesn’t do a great job. I think they’re more undergrad focused. But it would be nice if they had more- more clarity of like. you know. Maybe after 2 years they have to sit down with you and say, like, this is your your prospects. They don’t actually give you a firm offer, but like there's no room for you here, or something like that, or at least, or like, you know I don't know. Something so you like you don't waste your time right? sitting there, you know, you know, trying to push a boulder up a hill that will never go. You know.

00:48:09.240 --> 00:48:19.670

Moderator: I- I do wanna ask, and maybe the answer is that this is- this is an above the school level, and the school might not be able to do anything significant with this. But whether you have any recommendations specific to DEI?

00:48:27.180 --> 00:48:27.920

A.2.3: Hmm!

00:48:28.830 --> 00:48:32.600

A.2.1: I think that reco- I have some recommendations. But they're not feasible.

Moderator: Gotcha.

A.2.1: I- I think that those things need to be considered when hiring faculty at this university. I feel like we're at a place where you know the people who have been here have been here for 20 maybe some even 30 years, and their way of thinking is totally different than the way of thinking that we currently have today.

00:49:00.650 --> 00:49:04.749

A.2.1: And so we can talk a lot about our school thinking DEI is a priority. But again going back to the Supreme Court decision. And what we've seen in the past with the Nikole Hannah-Jones situation. It doesn't A.2.3er. Because, you know, everyone is right. Everyone has their own right to believe whatever they believe and what I've just personally learned like I like, I said. I've been here for 5 years, and I remember at a faculty meeting, one of the first faculty meetings that I attended, one of the tenured professors made a comment about, why are we trying to hire all this diverse talent? They’re gonna bring down like our talent, or something like- there was something to the effect, like..

00:49:51.520 --> 00:49:57.220

A.2.1: There were multiple people. At this faculty meeting. There were multiple people who heard him say this. And nothing was done about it. And when I brought it up to people about how [blind]omfortable I felt about what he said. It was just a “Oh, well, that’s just how he is. You know, he’s a tenured faculty. There’s really nothing we can do about that.” And so again, going back to.

00:50:15.760 --> 00:50:17.229

A.2.1: I don't.. again. Again. I do believe that DEI is important to the school.

00:50:21.340 --> 00:50:36.380

A.2.1: but until there's a systematic change in the school and the people that exist within the school. All of the work that they're doing does not A.2.3er, especially when you have people who are able to walk around like this unchecked like I've I've even had postdocs come to me and tell me that the chair of their department, in response to them getting a grant, “Oh, she just got that because diversity is a hot topic now, and they needed someone Black to award.”

00:50:49.710 --> 00:50:52.379

A.2.1: And so again my suggestion is not really feasible. Because again, you, you think about these are systemic issues that really require destruction of the system. And I don't think that the pharmacy is capable of actually destroying the system that has continuously fed it. So that's just what I have to say.

00:51:16.870 --> 00:51:19.240

Moderator: Yeah, that's fair.

325

00:51:19.340 --> 00:51:30.330

A.2.3: Yeah, it's spot on. And it kinda goes to like my whole point of you have to go above and beyond. So it's like your your work is beyond reproach. You can't say it's just- It's just because I’m a such and such a group. It's cause my research is awesome, right? Like that's that's why I'm here. That's what you- like and like. That's the pressure. Like, like, wellbeing, is like.. Cause I know that exists, is why I will push myself harder to try to rebut that, you know proactively right like I I'm like, I'm I try to do more. I try to be more into interdisciplinary. I try to go where other people don't, because..

331

00:52:02.360 --> 00:52:04.320

A.2.3: I know that's coming. And I know most of the time. But that's what people are gonna think when I when I sit there, and I know that won't fade right. It's just either like to systemic issues. And like you make your you make your contri- your drop in the bucket, and you you hope that like eventually it will.. And I- I do think it will get better with time. How long I don't know, but it's it's like, it's trending, it's just the gradient is so shallow. It doesn't like you. You still feel like we're not going anywhere. You probably are making progress it’s just not.. not as fast as I would like, and you know you just kind of accept that. But I- I- I think that's spot on. I don't think there's not much you can do other than have some of these guys, just age out and die.

A.2.1: That’s the sad truth.

00:52:57.460 --> 00:53:03.460

A.2.3: but yeah. yeah, I think that's that's kind of all I got.

00:53:04.940 --> 00:53:05.890

Moderator: okay.

00:53:06.040 --> 00:53:16.349

Moderator: Since we're kind of wrapping up here and getting towards the end, do you have any other thoughts or suggestions you'd like to share before we wrap up.

00:53:17.970 --> 00:53:27.849

A.2.3: Can you put the money thing down and underline and star it? Make sure that's top the top line? (Laughs) that covers a lot of crap. The

00:53:31.230 --> 00:53:35.099

A.2.3: no, I think I said, ever- all my piece.

00:53:35.490 --> 00:53:40.829

A.2.1: Yeah, II mean, I I'm kind of more familiar with the people that are doing this work, and I know how passionate they are about wellbeing so I think you know- I just I- I look at my experience here. And then I think about some of the things I've learned about wellbeing. And I think that it's it's just. It's such a huge thing. I think that we're gonna have to..

00:54:00.780 --> 00:54:14.150

A.2.1: We're like, well, being is important. Everyone should have a great experience at work. But then, when I see that there's so many things within academia that is fighting against, you know. That is- more power to y'all, that's all.

A.2.3: Oh, oh, more free food and more discounts. That's the other thing I want, like, cell phone discount. More free food.

00:54:32.380 --> 00:54:36.590

A.2.1: He might be losing services. He lost. Yeah.

00:54:36.970 --> 00:55:02.289

Moderator: Well, that was the last piece anyways. So I'll go ahead and drop the link in again for the post survey. It is also in your outlook calendar invite, and it's just a free space if you have anything else that you think of right after this meeting, or in the next few weeks that come up related to wellbeing That you wanna share.

A.2.1: alright. Thank you.

Moderator: Yeah, thank you.
